# Do Mixtures of Beads with Different Sizes Improve Wet Stirred Media Milling of Drug Suspensions?

**DOI:** 10.3390/pharmaceutics15092213

**Published:** 2023-08-26

**Authors:** Gulenay Guner, Mirsad Mehaj, Natasha Seetharaman, Sherif Elashri, Helen F. Yao, Donald J. Clancy, Ecevit Bilgili

**Affiliations:** 1 Otto H. York Department of Chemical and Materials Engineering, New Jersey Institute of Technology, Newark, NJ 07102, USA; gg357@njit.edu (G.G.); msm96@njit.edu (M.M.); ns895@njit.edu (N.S.); sme28@njit.edu (S.E.); 2 Drug Product Development, GlaxoSmithKline, Collegeville, PA 19426, USA; helen.f.yao@gsk.com (H.F.Y.); donald.j.clancy@gsk.com (D.J.C.)

**Keywords:** wet stirred media milling, bead mixtures, drug nanoparticles, breakage kinetics, machine learning, microhydrodynamic model

## Abstract

The impacts of bead sizes and bead mixtures on breakage kinetics, the number of milling cycles applied to prevent overheating, and power consumption during the nanomilling of drug (griseofulvin) suspensions were investigated from both an experimental and theoretical perspective. Narrowly sized zirconia beads with nominal sizes of 100, 200, and 400 µm and their half-and-half binary mixtures were used at 3000 and 4000 rpm with two bead loadings of 0.35 and 0.50. Particle size evolution was measured during the 3 h milling experiments using laser diffraction. An *n*th-order breakage model was fitted to the experimental median particle size evolution, and various microhydrodynamic parameters were calculated. In general, the beads and their mixtures with smaller median sizes achieved faster breakage. While the microhydrodynamic model explained the impacts of process parameters, it was limited in describing bead mixtures. For additional test runs performed, the kinetics model augmented with a decision tree model using process parameters outperformed that augmented with an elastic-net regression model using the microhydrodynamic parameters. The evaluation of the process merit scores suggests that the use of bead mixtures did not lead to notable process improvement; 100 µm beads generally outperformed bead mixtures and coarser beads in terms of fast breakage, low power consumption and heat generation, and low intermittent milling cycles.

## 1. Introduction

Nanoparticles have been widely used as a platform approach for bioavailability enhancement of poorly soluble drugs [1,2]. Either in liquid or solidified form, drug nanosuspensions can serve as drug delivery systems for various routes of administration, i.e., oral, parenteral, pulmonary, ocular, and dermal [3]. Liquid nanosuspensions can be used for parenteral liquid dosages [4]. In fact, long-acting parenteral suspensions are one of the niche applications of drug nanoparticles [5]. Wet stirred media milling (WSMM) is the most common technique both for the production of the marketed nano-formulated products and in academic studies [2]. This is not surprising, as WSMM is a reliable, reproducible, scalable, and solvent-free process that can be directly applied to any BCS Class II drug [2]. The process yield is usually high—typically, greater than 95% [6]. Drug nanoparticles tend to aggregate and grow during milling and storage [2,7]. Thus, various polymers with [8,9] and without surfactants [10,11] were used to mitigate this problem. Other challenges related to processing and manufacturing operations include long cycle times, high power and energy consumption, media wear, and ensuing product contamination [12,13]. To resolve these issues, the impacts of process conditions such as stirrer speed, bead loading, bead type, bead size, flow rate, and drug loading on breakage rate and cycle time [2,14,15], media wear [16,17], heat dissipation and temperature rise [18,19], power consumption, and operational costs [20,21] were investigated.

Despite the above-mentioned process studies, the pharmaceutical WSMM process is still one of the most time-consuming (hours to days) and energy-intensive processes (on the order of 1 MW/m^3^ power density) in the pharmaceutical industry, warranting process optimization studies. Note that any change in any WSMM variable can have pros and cons, which creates an interesting optimization problem when the trade-off of the impacts is considered. For instance, increasing stirrer speed and bead loading led to faster breakage of the particles and, therefore, shorter cycle times [14]. However, these conditions require higher power and energy consumption [14,21], which increases the cost of production [21] and bead wear/contamination [16,22], as well as heat generation and temperature rise as mechanical power is converted to heat [18,19]. Based on these findings, the existence of an optimal stirrer speed and bead loading combination is obvious.

Optimization strategies for the nanomilling of drugs also consider bead type and size. In WSMM, 50–90% *v*/*v* of the milling chamber volume is loaded with wear-resistant media such as yttrium-stabilized zirconia (YSZ) or cross-linked polystyrene (CPS) beads. While YSZ beads generally required higher specific energy consumption and led to higher heat generation and temperature rise than CPS beads, they achieved the same product fineness faster than CPS beads [20,21]. Noting that both YSZ and CPS beads have their inherent pros and cons, Guner et al. [20] optimized the WSMM process using a physical mixture of different bead materials, i.e., mixtures of polystyrene beads and zirconia beads.

Bead size is another important variable in WSMM. Milling beads provided by various suppliers tend to be narrowly sized and defined by their nominal sizes. If other process variables are chosen judiciously, small beads with nominal sizes of 50 and 100 µm can provide fast breakage kinetics and energy-efficient production with low wear and product contamination [16,17,23]. A comprehensive analysis of bead size effects [23] established a rationale for the selection of bead size for a given stirrer speed. According to Li et al. [23], an optimal bead size, which decreased from 400 µm at 1000 rpm to 50 µm at 4000 rpm, provided not only faster breakage but also better energy utilization. Smaller beads were also found to be more efficient for obtaining smaller particles by other groups [24,25,26], due to the increased number of beads and frequency of collisions [27]. At the beginning of the milling and depending on the feed drug particle sizes, large beads (300–800 µm) would be more beneficial to apply enough stress to break large particles. However, as milling progressed and finer drug particles were formed, these coarse beads became less effective due to their low capture efficiency of the sub-micron particles [23,28] and worse energy utilization [28]. In view of these considerations, pre-grinding of the feed suspension first using larger beads followed by wet milling with smaller beads [29] or dry pre-milling of the drug prior to WSMM offers optimization and may even be required due to severe clogging of the mill screen during the WSMM for coarse feeds of drug particles [30].

An alternative optimization strategy is to use mixtures of small and large beads in a single WSMM process. In fact, Kotake et al. [31] found that the so-called poly-sized media provide finer limestone particles compared to the single mono-sized media due to increased surface area. Patel et al. [32] investigated a possible synergistic effect by combining small (200 and 400 μm) and large (800 μm) beads and performed experiments in varying ratios of these beads while keeping all other WSMM parameters constant. They found that adding smaller beads to larger beads produced smaller barium sulfate and silica nanoparticles with lower energy consumption during milling. So, they concluded that the so-called mixed-media strategy enables the production of finer product particles with lesser energy consumption. Moreover, they claimed that this mixed media approach can lead to “huge savings” on grinding media costs since a small amount of costly small-sized media can be used in mixtures. Another claim was that the media wear could be improved by replacing smaller beads with coarser beads. However, none of these claims has been substantiated by experiments and/or analysis. Besides milling hard inorganic materials, these two studies [31,32] lack a comparison of poly-sized or mixtures of beads with small beads alone to understand if the advantage is purely due to the increased surface area of the beads or due to the combined advantage of large and small beads. Altun et al. [33] used a mixture of 1.5–2.5 mm and 3.0–4.0 mm alumina balls in dry stirred ball milling of limestone and compared their performance to those of the single ball size fractions in terms of milled particle size at various specific energy consumptions. The mixture performance lay in between the two individual ball size fractions, although some caveats were mentioned for the extremes of low and high energy consumption. While refs. [31,32] were the first studies to assess the feasibility of mixtures of beads with different nominal sizes, shortly referred to as bead mixtures hereafter, in the WSMM literature, they did not examine drugs or the impact of various stirrer speeds and bead loadings. Moreover, heat generation during the milling as well as bead wear aspects were not considered at all.

This study aims to provide a comprehensive analysis of the impacts of bead sizes and bead mixtures on the breakage rate, power consumption, and number of intermittent milling cycles during the WSMM of a drug (griseofulvin). To this end, narrowly-sized zirconia beads with 100, 200, and 400 µm nominal sizes as well as their half-and-half mixture combinations were used in milling at 3000 and 4000 rpm stirrer speeds with 0.35 and 0.50 fractional bead loadings. The pre-suspensions of 10% griseofulvin, 7.5% hydroxypropyl cellulose, and 0.05% sodium dodecyl sulfate were milled for 3 h, while the particle sizes were measured at certain intervals via laser diffraction along with mill outlet temperature and average power consumption. The breakage kinetics were analyzed by fitting an *n*th-order breakage kinetics model to the experimentally measured median drug particle sizes. Microhydrodynamic (MHD) parameters were calculated to examine the impact of process parameters and average bead sizes. Then, machine learning models were employed to predict the breakage kinetics in the test runs based on the MHD parameters and process parameters. Merit scores based on the inverse breakage rate constant, specific milling time, and number of intermittent milling cycles required to produce a median drug particle size of 200 nm, and power consumption were defined to compare the process performance at different processing conditions and enable us to assess the impacts of bead size and mixtures. Overall, the experimental-MHD analysis will allow us to test out if all the advantages of bead mixtures claimed in [32] for WSMM of inorganic materials are also applicable to WSMM of drugs. The capital cost of the beads and bead wear aspects are also considered to assess the potential benefits of the bead mixtures.

## 2. Materials and Methods

### 2.1. Materials

BP/EP-grade micronized griseofulvin (GF) was purchased from Letco Medical (Decatur, AL, USA). GF is a Biopharmaceutics Classification System (BCS) Class II drug and has a solubility of 14.2 mg/L at 37 °C [34]. Hydroxypropyl cellulose (HPC, L grade, Nisso America Inc., New York, NY, USA) was used as a non-ionic polymeric stabilizer, and sodium dodecyl sulfate (SDS, ACS grade, GFS chemicals, Columbus, OH, USA) was used as an anionic surfactant. Zirmil Y-grade yttrium-stabilized zirconia (YSZ) beads (Saint Gobain ZirPro, Mountainside, NJ, USA) with a 6000 kg/m^3^ density and 100, 200, and 400 µm nominal sizes were used. Their actual median sizes were 112, 194, and 403 µm, respectively, as measured by a laser diffraction particle size analyzer in the dry mode of dispersion (Helos/Rodos, Sympatec, NJ, USA). As can be seen from Table 1, these beads had a relatively narrow distribution with span values well below 1. In this paper, we refer to these beads received from the supplier as narrowly sized beads to distinguish them from their binary mixtures. The actual median sizes were used in the MHD calculations.

### 2.2. Experimental Methods

A pre-suspension of GF, HPC-L, and SDS was prepared using a shear mixer (Cat.# 14-503, Fisher Scientific, Pittsburgh, PA, USA) at 300 rpm for 2 h. The formulation was selected as 10% GF, 7.5% HPC-L, and 0.05% SDS with respect to 200 g of deionized (DI) water based on our prior investigations [21,35,36]. Although a lower concentration of HPC-L, such as 2.5%, could have been used for a stable GF suspension, that would lower the viscosity and power consumption. Very low power could not be measured reliably; hence, we purposefully selected 7.5% HPC-L to ensure physical stability while generating sufficiently high power needed by the microhydrodynamic analysis. The pre-suspensions were stored overnight at 8 °C after preparation and before milling. A Microcer wet stirred media mill (Netzsch Fine Particle Size Technology, LLC, Exton, PA, USA) was operated for 3 h with the parameters presented in Table 2, where the stirrer speed *ω*, bead loading *c*, and mass fraction of the beads with 100, 200, and 400 µm nominal bead sizes, i.e., *x*_100_, *x*_200_, and *x*_400_, were varied.

**Figure 1:**
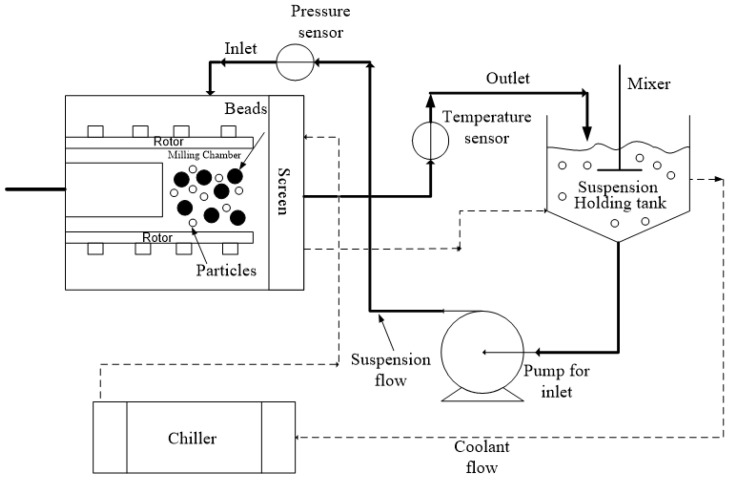
A schematic of the WSMM in recirculation mode of operation.

The processing conditions were based on our lab’s experience with WSMM of GF and different bead sizes [16,23,36]. We selected 3 h milling so that the WSMM either attained or approached the apparent grinding limit at the given set of conditions and produced the finest possible GF particles. The bead loading was calculated as the ratio of the true volume of the beads over the mill chamber volume *V*_m_ = 80 mL (*v*/*v*), which is filled by the recirculating drug suspension. Figure 1 presents a schematic of the WSMM in recirculation mode of operation. A peristaltic pump (Cole–Palmer, Master Flex, Vermont Hills, IL, USA) recirculated the drug suspension between the holding tank and the milling chamber at a volumetric flow rate *Q* of 126 mL/min. Different stainless-steel screens, whose openings are half the size of the smallest nominal bead size used in the mixtures, were used to keep the beads in the milling chamber. The setup was cooled using a chiller (Model M1-.25A-11HFX, Advantage Engineering, Greenwood, IN, USA) with a 20% *v*/*v* glycol–water mixture at about 8 °C. Until a total milling time of 3 h was achieved, additional intermitting milling cycles were applied when/if the temperature reached 35 °C [20,35]. The total number of milling cycles *N*_mc_ was recorded during the experiments. The average power consumption *P* was determined by dividing the cumulative energy consumption read from the control panel of the mill by the milling time. The average stirrer power per unit volume, *P*_w_, was calculated as *P*_w_ *= P*/*V*_m_. The power consumption during the stirring of the suspension in the absence of the beads *ε*_ht_ was found by the same method. The power consumption when there was no material in the mill (no-load) was obtained and subtracted during the calculation of *P*_w_ and *ε*_ht_.

Particle size distribution (PSD) of the drug suspensions at various milling times was determined by laser diffraction using the LS 13-320 Beckman Coulter instrument (Brea, CA, USA). The samples were taken from the mill outlet, where the temperature was measured, at certain time intervals (2^s^, s = 0, 1, 2, … 7) with the addition of 40 s, 24 min, 48 min, 96 min, 128 min, and 180 min. The final sample was taken from the holding tank, and all samples were measured with laser diffraction [37]. Before each measurement, a ~2.0 mL suspension sample was diluted with 5.0 mL of the respective stabilizer solution using a vortex mixer (Fisher Scientific Digital Vortex Mixer, Model No 945415, Pittsburgh, PA, USA) at 1500 rpm for one min. During measurements, polarized intensity differential scattering (PIDS) was maintained between 40% and 50%, while obscuration was maintained below 8%. The PSD and 10%, 50% (median size *d*_50_), and 90% passing sizes were provided by the software, which used the Mie scattering theory. The refractive indices of GF and water were taken as 1.65 and 1.33, respectively. Measurements were repeated four times, and the average and standard deviation (SD) of these measurements were determined. We used MATLAB’s pchip function, which is based on piecewise cubic Hermite polynomial interpolation, to determine the specific time for *d*_50_ to reach 0.2 µm (*t*_d50_) and then determined the associated number of intermittent milling cycles, *N*_d50_.

The apparent shear viscosities *μ*_L_ of the milled suspensions were measured using an R/S plus rheometer (Brookfield Engineering, Middleboro, MS, USA) with a water jacket assembly Lauda Eco (Lauda–Brinkmann LP, Delran, NJ, USA). A CC40 coaxial cylinder with a jacketed setup was used to impart a controlled shear rate on the samples from 0 to 1000 1/s in 60 s. The jacket temperature was kept constant at 25 ± 0.5 °C. The raw data were analyzed using the Rheo3000 software version 1, and the apparent shear viscosity at the maximum shear rate was taken. The density of the suspension was measured by weighing 35 mL of the milled suspension and dividing the mass of the suspension by its volume. The measurements were performed three times, and the average value was reported.

Run 19 was selected for morphology and solid-state analysis with scanning electron microscopy (SEM) and X-ray powder diffraction (XRPD). For the SEM with the Run 19 sample, a three-step centrifugation-dilution process was followed, as described in detail in ref. [38]: a droplet of the final diluted sample was placed on a carbon specimen holder and left in a desiccator for overnight drying. The BAL-TEC MED020 (BAL-TEC, Balzers, Switzerland) sputter coater was used to avoid charging. The imaging was performed with a JEOL JSM 7900F field emission scanning electron microscope (JEOL USA, Inc., Peabody, MA, USA). The as-received particles, whose SEM image was taken from our earlier work [23] for comparison purposes, were sputter coated and then imaged by a LEO 1530 SVMP (Carl Zeiss, Inc., Peabody, MA, USA). In both SEM imaging cases, the accelerating voltage was 5.0 kV. Prior to XRPD measurements, first, a portion of the nanosuspension in Run 19 was placed in a petri dish as a thin layer and left in a desiccator for 2 days, and then the dried film was ground with a mortar and pestle. The as-received GF and physical mixture were also mixed with a mortar and pestle to ensure a homogeneous blend. XRPD (PANanalytical, Westborough, MA, USA) was also used as described in ref. [38].

### 2.3. Theoretical Approaches

A microhydrodynamic (MHD) model was developed by Eskin et al. [39,40] to determine the mean velocity of bead oscillations in well-mixed slurries using the kinetic theory of granular flows and fundamental granular energy balance [41]. The main modifications to the MHD model, implemented by Bilgili and Afolabi [42], include the use of experimentally measured power consumption and the addition of a term that accounts for the power spent on shearing the slurry at the same shear rate without the beads *ε*_ht_. While only the key equations are presented here, all assumptions and derivation steps can be found in the mentioned literature. The power per unit volume of slurry *P*_w_ inside a stirred mill equals the sum of three components:

(1) $$P_{w}=\varepsilon_{\mathrm{visc}}+\varepsilon_{\mathrm{coll}}+\varepsilon_{\mathrm{ht}}$$

where *ε*_visc_ is the energy dissipation rate due to both liquid–bead viscous friction and lubrication, and *ε*_coll_ is the energy dissipation rate due to partially inelastic bead–bead collisions. Inserting the respective expressions for *ε*_visc_ and *ε*_coll_, Equation (1) becomes

(2) $$P_{w}=\frac{54\mu_{L}c\theta R_{\mathrm{diss}}}{D_{b}^{2}}+\frac{12}{D_{b}\sqrt{\pi }}\left(1-e^{2}\right)g_{0}c^{2}\rho_{b}\theta^{3/2}+\varepsilon_{\mathrm{ht}}$$

in which *μ*_L_ is the apparent shear viscosity of the milled suspension, *c* is the bead volumetric concentration (volume fraction), *θ* is the granular temperature defined as one third of the bead-milled suspension relative mean-square velocity, *R*_diss_ is the effective drag coefficient (see Equation (A1)), *D*_b_ is the median size of the beads, *e* is the restitution coefficient for the bead–bead collisions (0.76 for YSZ beads [43]), *ρ*_b_ is the density of the beads (6000 kg/m^3^), *c* is the volumetric bead loading, and *g*_0_ is the radial distribution function at contact. The Lun model [44] was used for *g*_0_ (Equation (3)), as it exhibited a better predictive capability of the microhydrodynamics and breakage kinetics [45], where *c*_lim_ is the packing limit and equals 0.63 [46].

(3) $$g_{0}={\left[1-{\left(c/c_{\lim}\right)}^{1/3}\right]}^{-1}$$

All parameters and variables in Equation (2) are known or experimentally measured except for the granular temperature, which was determined using MATLAB’s fsolve function. While all equations and parameters are reported in Appendix A, only the MHD parameters that were used for breakage rate predictions are presented here. These parameters are the maximum bead contact pressure at the center of the contact circle *σ*_b_^max^, radius of the contact circle *α*_b_, average frequency of drug particle compressions *a*, and the pseudo-energy dissipation rate for the drug particles $\Pi \cdot \sigma_{y}$.

(4) $$\sigma_{b}^{\max}=\frac{3F_{b}^{n}}{2\pi \alpha_{b}^{2}}$$

(5) $$\alpha_{b}={\left[\frac{3\left(1-\eta_{b}^{2}\right)}{4Y_{b}}R_{b}F_{b}^{n}\right]}^{1/3}$$

(6) $$a=p\nu =11.64\frac{c^{2}g_{0}}{\sqrt{\pi }\left(1-c\right)}{\left[\frac{\rho_{b}\left(1-\eta_{b}^{2}\right)}{Y_{b}}\right]}^{2/5}\frac{R_{p}}{R_{b}^{2}}\theta^{9/10}$$

(7) $$\Pi \cdot \sigma_{y}=4.46\frac{c^{2}g_{0}}{\pi^{5/2}\psi }{\left(\frac{Y_{b}}{1-\eta_{b}^{2}}\right)}^{18/15}{\left(\frac{Y^{*}}{Y_{p}}\right)}^{1/3}\rho_{b}^{4/5}\frac{R_{p}}{R_{b}^{2}}\theta^{13/10}$$

In Equations (4)–(7), *F*_b_^n^ is the average maximum normal force during the collision of two elastic beads (see Equation (A7)), *Y*_b_ and *ɳ*_b_ are the Young’s modulus and Poisson’s ratio of the bead material (0.2 and 200 GPa for YSZ beads [47]), *R*_b_ is the bead radius, *p* is the probability of a single drug particle with radius *R*_p_ (the initial median radius: 5.7 µm) being caught between beads (see Equation (A8)), and *ν* is the frequency of single-bead oscillations (see Equation (A6)). In the MHD calculations, *R*_b_ was taken as half the measured median size *d*_50_ of the beads (Table 1). For the 50–50% mixtures of the beads with different bead sizes, an average *d*_50_ of the respective nominal bead sizes was used because the measured sizes of the beads were close to their nominal values. The parameters *φ*, *σ*_y_, Y*, *Y*_p_, and *η*_p_ represent the volume fraction of the drug particles in the suspension (0.061), contact pressure in a drug particle captured when the fully plastic condition was reached, reduced Young modulus of the bead–drug particle contact (see Equation (A9)), Young modulus of the drug particles, and Poisson’s ratio of the drug particles. The values of *Y*_p_ and *η*_p_ were taken from ref. [48] as 11.5 GPa and 0.3. For the sake of completeness, all MHD parameters calculated are reported in Table S1.

The following *n*th-order breakage kinetics model derived in ref. [35] was used to fit the timewise evolution profile of the experimentally measured median particle sizes:

(8) $$d_{50}\left(t\right)=d_{\lim}+{\left[{\left(d_{50,0}-d_{\lim}\right)}^{1-n}-\left(1-n\right)kt\right]}^{1/\left(1-n\right)}$$

wherein *d*_50,0_ is the initial median size, *d*_lim_ is the limiting median size, and *k* is the breakage rate constant. The Marquardt–Levenberg optimization algorithm in Sigmaplot (Version 12.5) was used to fit the log-transformed experimental median sizes at various times, and *d*_lim_, *n*, and *k* were estimated. In the fitting, a constraint was placed on the limiting particle size to ensure that it was smaller than the final median particle size [21].

A relationship between the estimated parameters of the *n*th-order model (*k*, *n*, and *d*_lim_) and the calculated MHD parameters (*σ*_b_^max^, *α*_b_, *a*, and $\Pi \cdot \sigma_{y}$), as well as the process parameters (*ω*, *c*, *x*_100_–*x*_200_–*x*_400_), were sought using machine learning algorithms. Google Colab was used for this analysis, where the sklearn package of Python was utilized. Most of the models were used to perform regression on all responses at the same time, and the total of the root mean squared errors (RMSE) of all responses was obtained. On the other hand, gradient boosting was not suitable for performing regression on multiple responses, so the MultiOutputRegressor command, which performs one regression per response, was used, and the total RMSE was reported as well. For the model selection, the models were calibrated using the training set, which consists of 24 experiments with full factorial DOE (refer to Table 2). They were also tested using three separate runs at the average conditions of the design space with the individual bead sizes. Leave-one-out cross validation was used in the training set to assess the prediction capability of the models, and RMSEs were reported. Finally, the models were selected as the ones that gave the lowest test RMSE. Please note that experimental data (power and viscosity) were used to estimate the MHD parameters, which are the predictors in the test set for MHD-based prediction.

In order to rank-order the performance of the WSMM process with different milling conditions and assess the impact of bead mixtures vs. nominal single-sized beads, merit scores were defined as motivated by our earlier work [20,21]. Normalized values of either the inverse breakage rate constant 1/*k* or the specific time to reach a *d*_50_ = 0.2 µm (*t*_d50_) (as rough measures of cycle time), the power *P*, and the number of intermittent milling cycles for *t*_d50_ (*N*_d50_) were calculated as follows:

(9) $$\bar{1/k}=\left[1/k-{\left(1/k\right)}_{\min}\right]/\left[{\left(1/k\right)}_{\max}-{\left(1/k\right)}_{\min}\right]$$

(10) $$\bar{t_{d50}}=\left(t_{d50}-t_{d50,\;\min}\right)/\left(t_{d50,\;\max}-t_{d50,\;\min}\right)$$

(11) $$\bar{P}=\left(P-P_{\min\;}\right)/\left(P_{\max}-P_{\min}\right)$$

(12) $$\bar{N_{d50}}=\left(N_{d50}-N_{d50,\min\;}\right)/\left(N_{d50,\max}-N_{d50,\min}\right)$$

The merit scores were calculated in two different ways using the normalized values of either 1/*k* or *t*_d50_ as follows:

(13) $$\mathrm{Merit}\;\mathrm{Score}=100/10^{(\bar{1/k\;}+\bar{P}+\bar{N_{d50})}/3}$$

(14) $$\mathrm{Merit}\;\mathrm{Score}=100/10^{(\bar{t_{d50}\;}+\bar{P}+\bar{N_{d50})}/3}$$

The merit scores theoretically range from a minimum of 10 and a maximum of 100; however, practically, they may not exceed 80 because highly energetic WSMM processes with higher *P* and *N*_d50_ values tend to have shorter cycle times (lower 1/*k* or *t*_d50_).

## 3. Results and Discussion

Just for the sake of labeling, the nominal bead sizes and the average sizes for the bead mixtures are indicated in the figures and the text, while the actual median bead sizes from the laser diffraction (refer to Table 1) were used for the MHD calculations. In this context, the average sizes in the bead mixtures were labeled with 100, 150, 200, 250, 300, and 400 µm for the 100-0-0, 50-50-0, 0-100-0, 50-0-50, 0-50-50, and 0-0-100 mass fractions of the 100, 200, and 400 µm narrowly-sized beads, respectively.

### 3.1. Breakage Kinetics, Power Consumption, Heat Generation, and Processing Issues

**Figure 2:**
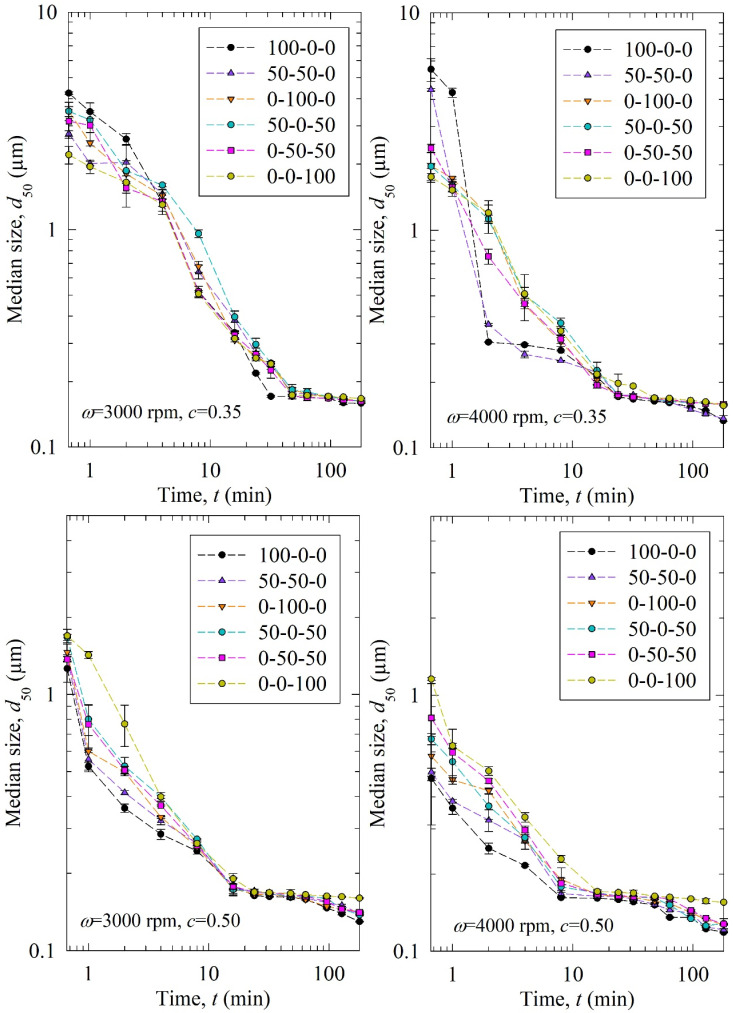
Timewise evolution of the median drug particle size *d*_50_ during 180 min of milling with various mass fractions of 100-00-400 µm narrowly sized beads at different stirrer speeds *ω*–bead loadings *c*.

Figure 2 shows the time-wise evolution of the median particle size *d*_50_; each subfigure is for a different stirrer speed *ω*–bead loading *c* pair and each curve in a subfigure is for a different bead size *D*_b_ (narrowly sized beads and binary bead mixtures). For the sake of completeness, Figures S1 and S2 in the Supplementary Materials show the time-wise evolution of 10% and 90% passing sizes *d*_10_ and *d*_90_, respectively. The percentages of the three narrowly sized beads are given in the legend. The feed GF particles had the characteristic sizes of *d*_10_ = 4.30 µm, *d*_50_ = 11.4 µm, and *d*_90_ = 23.5 µm, while the GF particles had a *d*_50_ below 200 nm upon 3 h milling at all process conditions studied, signifying the drastic size reduction during the WSMM. A general observation from Figure 2 is that the decrease in the median size of the drug particles was nearly monotonic within experimental accuracy. This finding suggests that breakage is the dominant particle change mechanism, whereas aggregation did not occur to a significant extent. If aggregation were dominant, *d*_50_ would have increased during the milling upon the formation of finer particles (see, e.g., [49,50]). Also, *d*_50_ approached an apparent limiting particle size in all process conditions, which is consistent with the nanomilling of other drugs [51,52].

Stirrer speed, bead loading, and bead mixture compositions are the variables investigated in this study. According to Figure 2, upon an increase in the stirrer speed *ω* and the bead loading *c*, particle breakage occurred faster, as indicated by a smaller *d*_50_ at a certain milling time. As the profiles are close to each other, the differences between the six curves for the different bead sizes/mixtures may be hard to discern. Therefore, we fitted the *n*th-order breakage kinetics model and compared the breakage rate constant *k*. The fitted parameters and statistics are presented in Table 3. Overall, the *n*th-order model fitted most experiments well with an average adjusted R^2^ above 0.95, and only one run had it below 0.90: 0.87 in Run 13. The mean and standard deviation of *d*_lim_ were found to be 0.125 ± 0.026 µm. The average *n* was found to be ~2 similar to previous studies [20,35], but with a higher standard deviation of 0.36. The difference might be due to different bead sizes/mixtures, and drugs used in these studies. In addition, the usage of bead mixtures might have caused a different order of breakage kinetics from two because coarser beads were more effective in breaking only the coarse drug particles initially present (typically about >10 µm), whereas small beads were more effective for breaking all particles below 5 µm, especially those in the colloidal size domain. This argument is based on the notion of an optimal bead size:feed particle size ratio of 20:1, recommended for WSMM of hard materials like quartz [53], as well as on the median sizes of the feed GF particles and the narrowly-sized beads.

**Figure 3:**
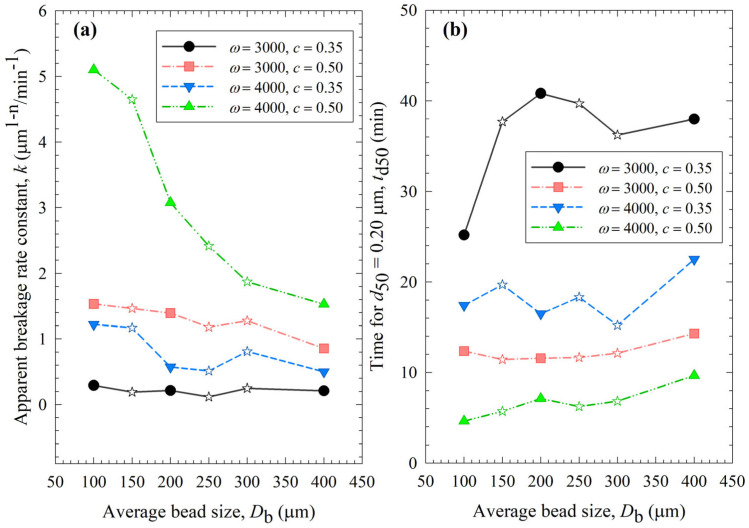
Impact of the average bead size on (**a**) the breakage rate constant *k* and (**b**) the time needed for the median particle size to reach 0.20 µm *t*_d50_ for various stirrer speeds *ω*–bead loadings *c*. Average bead sizes of 150, 250, and 300 µm correspond to 50–50% *w*/*w* mixtures of 100–200 µm, 100–400, and 200–400 µm beads, respectively, and those are shown with star symbols.

Figure 3 presents the impact of process conditions on the breakage rate constant *k* and a specific time for the median size to reach 0.2 µm *t*_d50_. A higher *k* value or a lower *t*_d50_ value is an indication of faster breakage, and their trends are usually opposite each other. It is worth mentioning that the determination of *k* is based on all milling times, whereas that of *t*_d50_ is affected by a few time points via interpolation in the neighborhood of 0.2 µm. So, they are not expected to correlate perfectly. The slopes were the steepest for the 4000 rpm, 0.5 bead loading case (Run 19), which exhibited the fastest breakage: the *k* for the smallest beads was 3.3 times the *k* for the largest beads. The statistical analysis in Section S2 indicated the following ranking of the parameters in terms of their statistically significant influence on *k*: stirrer speed (positive correlation) > bead loading (positive correlation) >> average bead size (negative correlation). In general, the data presented in Figure 3 and Table 3 suggest that (i) higher *k* and lower *t*_d50_ occurred at higher stirrer speed and/or higher bead loading; (ii) with a few exceptions, *k* tended to decrease and *t*_d50_ tended to increase when the average size of the beads was increased; and (iii) most *k* and *t*_d50_ values for the various mixtures were bound between those of the respective narrowly sized beads, or when they were outside these bounds, the deviations were rather small, typically less than ~20%. The upshot of these results is that the stirrer speed and the bead loading had a stronger impact on the breakage kinetics than the average bead size; the bead mixtures did not exhibit significant synergistic improvement. The 100 µm beads alone outperformed coarser beads and the bead mixtures in terms of faster kinetics.

**Figure 4:**
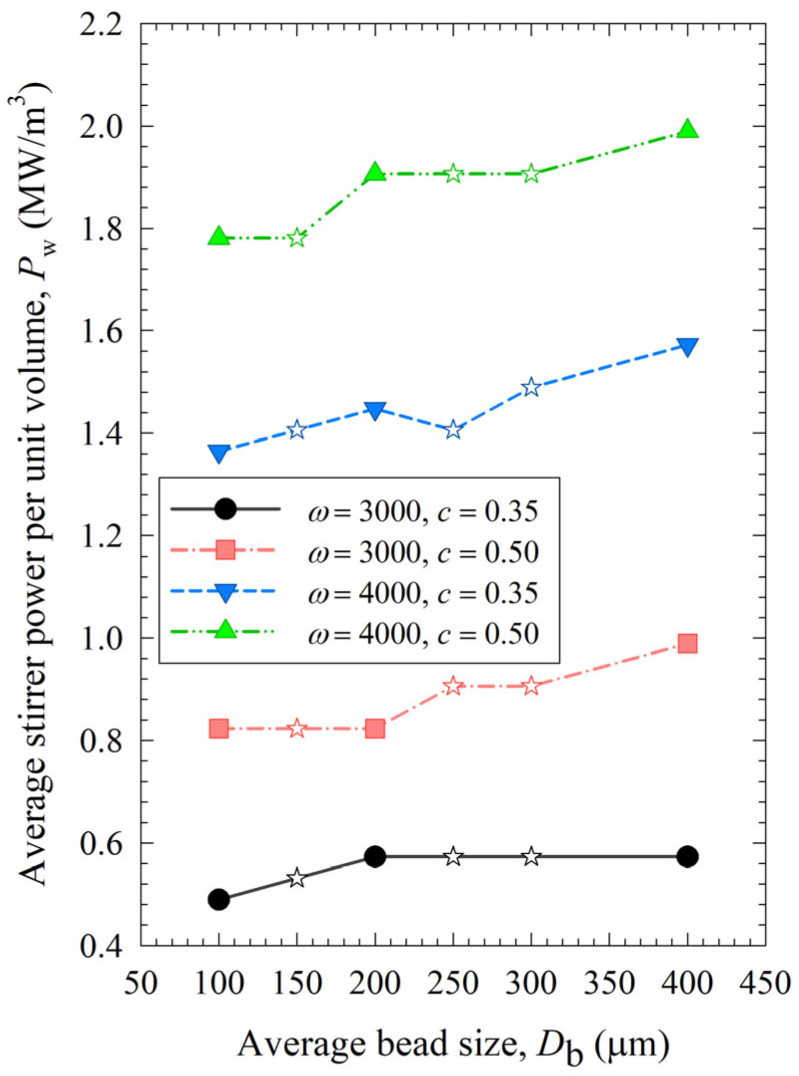
Impact of the average bead size on the average power consumption for each *ω*–*c* pair. Average bead sizes of 150, 250, and 300 µm correspond to 50–50% *w*/*w* mixtures of 100–200 µm, 100–400, and 200–400 µm beads, respectively, and those are shown with star symbols.

The impacts of process conditions on power consumption are illustrated in Figure 4. The curves were rather flat, signifying that the bead size impact was not as influential as the stirrer speed and the bead loading. Still, coarser beads (200 and 400 µm) require more power to operate than 100 µm beads, which makes smaller beads even more advantageous. An increase in either stirrer speed or bead loading led to an increase in power, which is in line with established correlations [54,55]. Moreover, most power values for the various mixtures were bound between those of the respective narrowly sized beads, or when they were outside these bounds, the deviations were rather small, typically less than ~20%. The significance of the parameters that affect power consumption was investigated with regression analysis, as shown in Section S3 of the Supplementary Materials. In addition to stirrer speed, bead loading, and bead size, viscosity is also included as a predictor. The instantaneous power readings decreased during milling, as discussed in ref. [19], due to the decrease in viscosity with decreasing particle size and increasing mill temperature. While the power consumption was positively and significantly correlated with the stirrer speed and the bead loading, the bead size effect was much less significant, and the viscosity effect was not statistically significant in this data set due to its small variation (Section S3). In addition, there is interaction between these predictors. For instance, the lower viscosity of a nanosuspension was partly related to its smaller particles, which were produced when higher stirrer speeds and bead loading conditions were used in the milling. Therefore, the impact of viscosity on power consumption could not be decoupled from stirrer speed/bead loading effects with a data set like this.

The increase in power with increasing stirrer speed, bead loading, and bead size also led to an increase in the number of milling cycles *N*_mc_ (during the 3 h milling), as can be seen in Table S2. As most of the power was converted to heat, the heat generation rate and temperature rise were higher when the power was higher, thus requiring more frequent shutdowns for cooling without milling (intermittent milling). Numerous cycles had to be applied, especially at 4000 rpm runs, which were more frequent compared to a prior heat transfer study [19], due to prolonged milling and a lower value of the maximum temperature allowed in the current study: 45 °C in [19] vs. 35 °C here. Table S2 shows that 100 µm beads outperform all other narrowly sized beads and bead mixtures (lowest *N*_mc_) under all processing conditions. The bead mixtures did not provide any synergistic benefit in reducing *N*_mc_. Shutting down a mill for cooling without milling (*N*_mc_ > 1) is undesirable for pilot and commercial-scale operations. However, this intermittent milling is unavoidable if one wants to keep the temperature under control in small-scale milling equipment under the highly energetic processing conditions explored here. The main reason for this is the inadequate bulk convective cooling provided by the recirculating suspension, as the thermal inertia of the suspension batch placed in the holding tank of a small-scale mill is much smaller than that in pilot-commercial-scale equipment. Of course, the relatively low cooling capacity of the particular chiller in our mill also contributed to the need for intermittent milling.

Finally, we want to mention a processing issue, hydraulic packing, and associated partial clogging of the mill screen, during the early milling times up to 8–16 min typically when 100 µm beads and their mixtures were used, especially at a loading of *c* = 0.35. The clogging issue became less notable and of shorter duration for the mixtures of the 100 µm beads with the 200 and 400 µm beads, although it was not eliminated. No clogging was observed for the 200 and 400 µm beads under any process conditions. While the drug suspension was passing through the mill at 126 mL/min, it applied drag forces on the beads, which might have overcome the turbulent motion of the beads, thus forcing them to pack around the mill’s screen (hydraulic packing). This phenomenon is known to be more prevalent with smaller beads and higher suspension flow rates and viscosities [56]. Hydraulic packing becomes severe and causes process shut-down if the pressure rise becomes too high and product flow is disrupted significantly. As the small beads (100 µm) were used along with the smallest screen opening size, i.e., 50 µm, and they were not as effective as the larger beads in nipping the coarse drug particles initially present, hydraulic packing of the 100 µm beads led to partial clogging of the screen. We managed the hydraulic packing by changing the flow direction of the pump to reverse and reverse-back the flow of the suspension several times. This practice disrupted the hydraulic packing and allowed sufficient time for the reduction of the drug particle size to a sufficiently small value, concomitantly reducing the viscosity, which prevented further clogging during prolonged milling. Obviously, this practice may not be very desirable in an industrial setting. Despite this issue during the initial milling, upon further milling, drug particles became smaller; 100 µm beads caught up quickly and provided even faster breakage towards the end of 3 h of milling, as compared with coarser beads (400 µm) (Figure 2).

### 3.2. Further Characterization of the Milled Drug Suspensions and Particles

**Figure 5:**
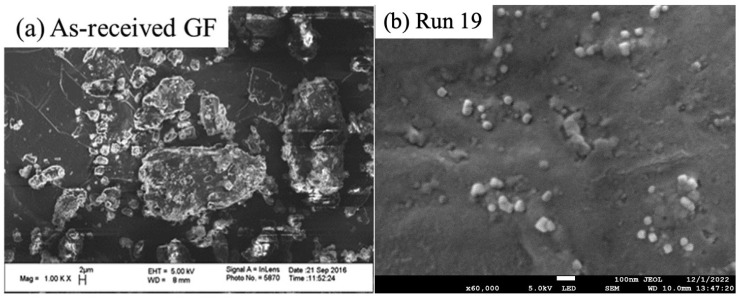
SEM images of (**a**) as-received GF particles (Magnification: ×1000, EHT: 5.00 kV, WD: 8 mm, Marker size: 2 µm) and (**b**) milled GF particles in Run 19 (Magnification: ×60,000, EHT: 5.0 kV, WD: 10 mm, Marker size: 100 nm). Figure 5a was adapted with permission from Ref. [23], 2017, Elsevier.

The drastic particle size reduction caused by milling was also seen in the SEM images (Figure 5), where the first one shows the as-received GF particles and the second one shows the particles in the milled suspension in Run 19, which provided the smallest particle sizes. The sizes of the particles in the SEM images match the laser diffraction measurements, and non-aggregated individual particles can be seen, indicating the suspensions were well stabilized. This is not surprising, as the HPC–SDS combination was shown to have a synergistic action on the stabilization of GF suspensions [36]. This combination was purposefully selected in this study to minimize any confounding effects from aggregation on the breakage kinetics.

**Figure 6:**
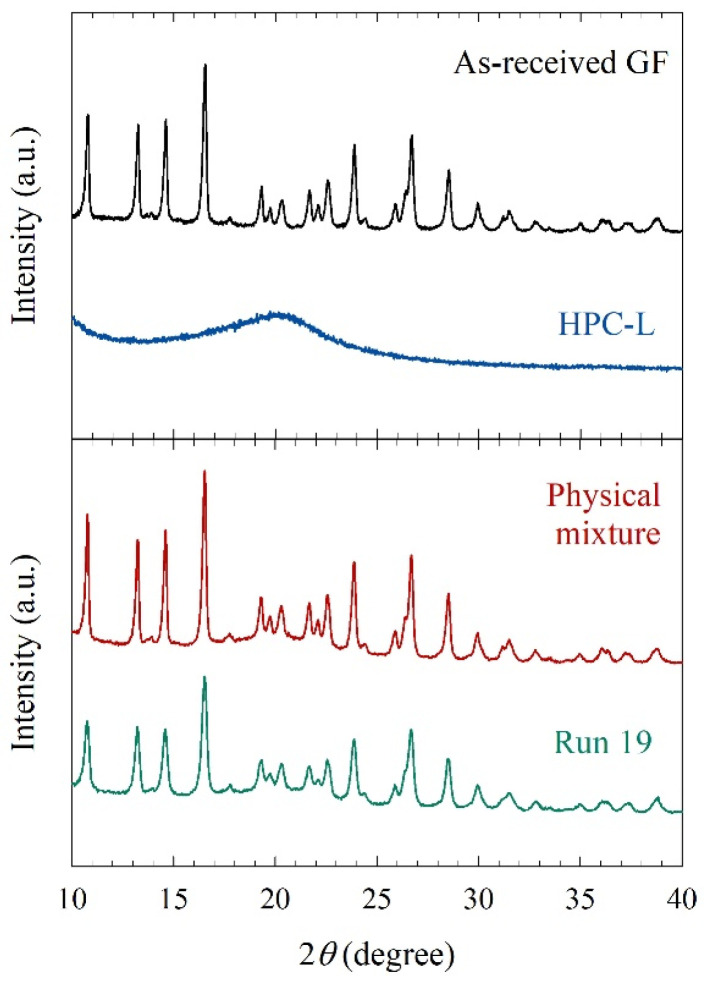
XRPD diffractograms of as-received GF, HPC-L, physical mixture, and the dried powder of the nanosuspension in Run 19.

Mechanical stresses that cause particle breakage during nanomilling may also cause polymorphic changes or amorphization of drugs. Figure 6 compares the XRPD diffractograms of the as-received GF, the physical mixture of the formulation, and the overnight dried milled particles (Run 19). As-received GF exhibited diffraction peaks characteristic of a crystalline material, whereas HPC exhibited a broad halo pattern, characteristic of an amorphous polymer. The dried, milled GF particles in Run 19 had characteristic peaks at the same diffraction angles but slightly depressed peaks as compared with the physical mixture and the as-received GF. This can be attributed to better surface coverage of nanoparticles by HPC and defect formation during WSMM. Overall, milling was intense enough to cause drastic particle size changes but did not cause any undesired solid-state changes.

**Figure 7:**
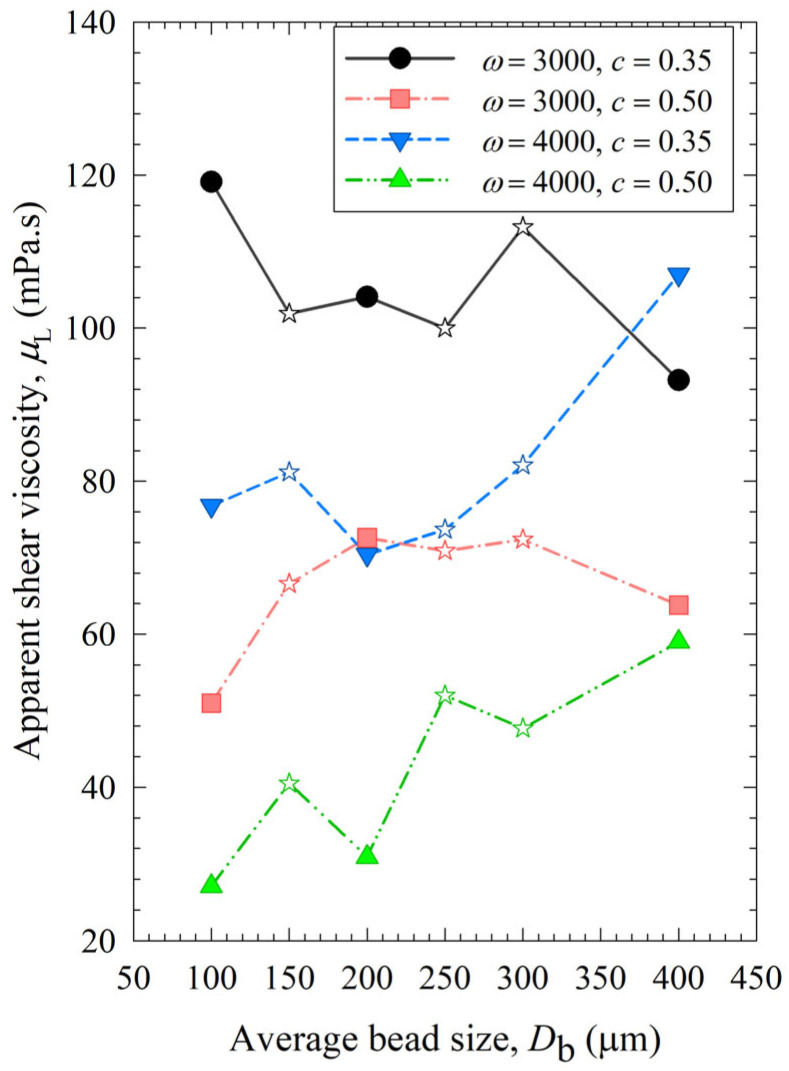
Impact of the average bead size on the apparent shear viscosity of the milled suspension for each *ω*–*c* pair.

Figure 7 shows that similar to power, the viscosity of the milled suspensions was lower when smaller beads were used. Besides, it decreased with higher bead loading and stirrer speed, as smaller drug particles in the suspensions account for the lower viscosity [22]. Additional analysis was done for viscosity dependency on the stirrer speed, bead loading, and average bead size (Section S4 in Supplementary Materials), which has the opposite trends to those observed for breakage rate constant *k* as shown in Section S2. This supports our hypothesis that viscosity was affected by particle size and positively correlated with the final median size *d*_50_ (Section S5 of the Supplementary Materials).

### 3.3. Microhydrodynamic (MHD) Analysis of the Impact of the Processing Conditions

The MHD model was formulated with the major assumption that the beads are spherical and monodispersed [39,40]. Although the as-received beads with nominal sizes of 100, 200, and 400 µm have a relatively narrow distribution (refer to Table 1), the bead mixtures automatically have much wider distributions due to the 50–50% combination of the respective bead sizes. Hence, the MHD model is expected to be more accurate for the narrowly sized beads than for the bead mixtures. To correlate the breakage rate to the MHD parameters for the case of bead mixtures, the average of the median bead sizes of the relevant narrowly sized beads in the 50–50% binary mixtures was used in the calculations as a rough first approximation.

**Figure 8:**
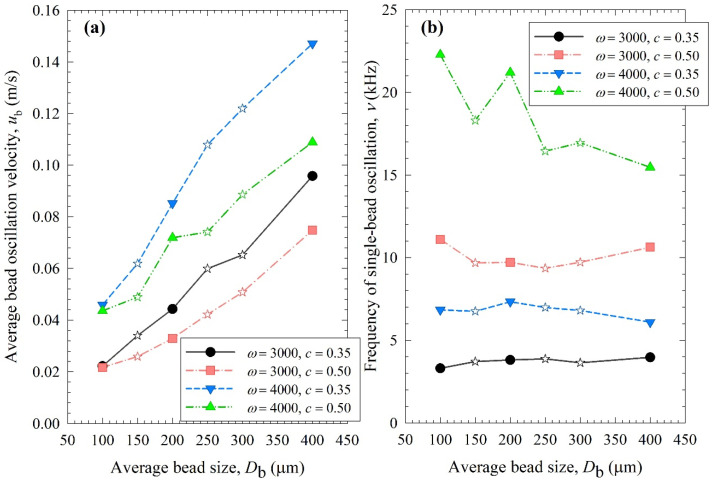
Impact of the average bead size on (**a**) the average bead oscillation velocity *u*_b_ and (**b**) the frequency of the single-bead oscillation *ν* for each *ω*–*c* pair. Average bead sizes of 150, 250, and 300 µm correspond to 50–50% *w*/*w* mixtures of 100–200 µm, 100–400, and 200–400 µm beads, respectively, and those are shown with star symbols.

**Figure 9:**
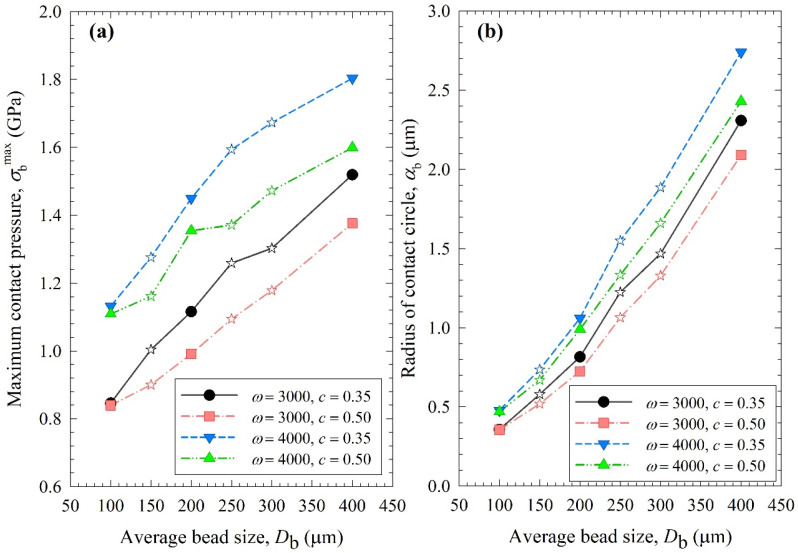
Impact of the average bead size on (**a**) the maximum contact pressure *σ*_b_^max^ and (**b**) the radius of contact circle *α*_b_ for each *ω*–*c* pair. Average bead sizes of 150, 250, and 300 µm correspond to 50–50% *w*/*w* mixtures of 100–200 µm, 100–400, and 200–400 µm beads, respectively, and those are shown with star symbols.

**Figure 10:**
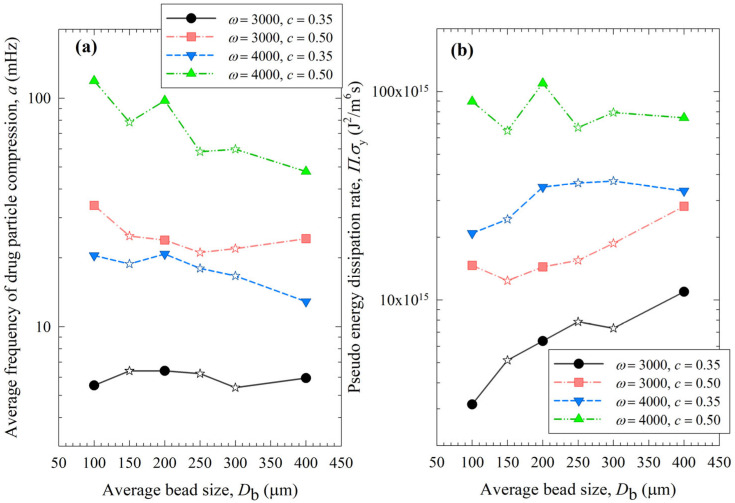
Impact of the average bead size on (**a**) the average frequency of drug particle compressions *a* and (**b**) the pseudo energy dissipation rate $\Pi \cdot \sigma_{y}$ for each *ω*–*c* pair. Average bead sizes of 150, 250, and 300 µm correspond to 50–50% *w*/*w* mixtures of 100–200 µm, 100–400, and 200–400 µm beads, respectively, and those are shown with star symbols.

Figure 8, Figure 9 and Figure 10 show the change in each MHD parameter with increasing average bead size, where each figure is for a different MHD parameter couple, and different ω–c pairs are shown with different lines in all subfigures. All MHD parameters are also reported in Table S1. Let us first examine the microhydrodynamic data in Figure 8, Figure 9 and Figure 10 and Table S1 (for *θ*) and assess the impacts of stirrer speed ω and bead loading *c*. For all bead mixtures, an increase in ω led to a higher power *P*_w_ (see Figure 4); more frequent and energetic fluctuating motion of the beads (as signified by higher ν, *u*_b_, and *θ*, respectively); higher stress intensity, bead deformation, and energy dissipation rate associated with drug particle deformation (as signified by higher σ_b_^max^, *α*_b_, and $\Pi \cdot \sigma_{y}$, respectively); and a higher frequency of drug particle compression events (higher *a*). As all these effects favor particle breakage, they provide a physical, mechanistic basis as to why higher ω led to higher *k* and a lower *t*_d50_ (refer to Table 3).

On the other hand, two counteracting effects of higher *c* were noted: while higher frequencies of bead oscillations ν and of drug particle compressions *a* occurred, the fluctuating kinetic energy of the beads and the stress intensity of the bead–bead collisions were lower, as signified by lower *θ* and σ_b_^max^ (also lower *u*_b_ and *α*_b_). These changes resulted from the higher number concentration, radial distribution function at contact, and higher dissipation rate due to drag. Note that the relative increase in ν and *a*, favorable for breakage kinetics, was much more pronounced than the relative decrease in *θ*, σ_b_^max^, *u*_b,_ and *α*_b_, unfavorable for breakage kinetics. Moreover, drugs are softer and easier to break than most inorganic materials, thus obviating the need for very high stress intensities (σ_b_^max^ and $\Pi \cdot \sigma_{y}$) for breakage. Hence, the favorable impact of a higher *c* was dominant, which explains why a higher *c* led to a higher *k* and a lower *t*_d50_ (refer to Table 3). These findings are in line with previous microhydrodynamic studies with different process parameters, bead sizes, and drugs [14,23,35,45].

Let us now examine the MHD effects of average bead size *D*_b_. Interestingly, we also note two counteracting effects of bead size: On one hand, an increase in bead size led to higher stress intensity with more bead deformation (higher σ_b_^max^ and *α*_b_ in Figure 9), which resulted from higher fluctuating kinetic energy *θ* (see Table S1) and bead oscillation velocity *u*_b_ (Figure 8a). The pseudo-energy dissipation rate (Figure 10b) was higher for the bigger beads, except for the highest energetic condition, as a result of contrary trends in stress intensity (*σ*_b_^max^)–stress frequency (*a*). All of these changes can be attributed to the higher power *P*_w_ (see Figure 4) and the lower bead number concentration associated with the coarser beads. Up to this point, one may argue that drug particle breakage would be faster upon the use of coarser beads as the aforementioned changes in the MHD parameters favor particle breakage. On the other hand, while the frequency of bead oscillations ν was somewhat similar for 3000 rpm runs, it increased when smaller beads were used for 4000 rpm runs (Figure 8b). Most importantly, while the average frequency of drug particle compressions *a* was similar for all bead sizes in the lowest energetic case, it was higher for the smaller beads in the *ω* = 3000 rpm–*c* = 0.5 bead loading case, and the difference became more pronounced at 4000 rpm runs (Figure 10a). This effect of the smaller beads, especially on *a*, favors faster breakage. As discussed above, one would argue that for relatively soft and brittle materials like drugs, a high stress intensity σ_b_^max^ is not needed provided it is above a low threshold value, which makes *a* the most important microhydrodynamic parameter. In fact, earlier MHD studies established that *a* had the strongest impact on and was most strongly correlated to the breakage rate constant *k* [35,45]. Even a cursory look at Figure 3 and Figure 8–10 reveals that the general trends as to how *k* changed with *ω*, *c*, and *D*_b_ were emulated only by how *ν* and *a* changed with *ω*, *c*, and *D*_b_.

When the MHD parameters of narrowly sized beads and bead mixtures were compared, those of the bead mixtures usually fell around the trendlines of the narrowly sized beads (Figure 10). However, some deviations from the trendlines were observed, especially for *a* and $\Pi \cdot \sigma_{y}$ at the higher energetic runs (Figure 10). This is partly due to the nonlinearity of *a* and $\Pi \cdot \sigma_{y}$ trends with bead sizes, where *a* was shown to have an exponential decay-like relationship with bead size in ref. [16]. Most importantly, these deviations originated from the oversimplification of treating the bead mixture as an equivalent monodispersed bead with the calculated average bead size. In the MHD model, there is no distinction between different types of collisions of beads with different sizes and associated radial distribution functions. The radial distribution function was assumed to be a function of bead concentration alone for monodispersed spheres, but not the bead sizes. It is also possible that beads of different sizes have different granular temperatures [57]. Unfortunately, the existing MHD model does not treat these different aspects of bead mixtures. New, expanded MHD models must consider different bead sizes for accurate prediction of the bead mixtures. In the literature for two-phase gas–solid flows, mixtures of different particle sizes have been considered for polydisperse powders, and different values of the radial distribution function g_0_ were determined [57,58]. For example, in the context of a binary mixture of fine–coarse particles, different g_0_ expressions have been formulated for the various types of particle collisions, i.e., fine–coarse, fine–fine, and coarse–coarse. Such approaches must be adopted in advancing the MHD model for bead mixtures.

### 3.4. Breakage Kinetics Predictions

**Figure 11:**
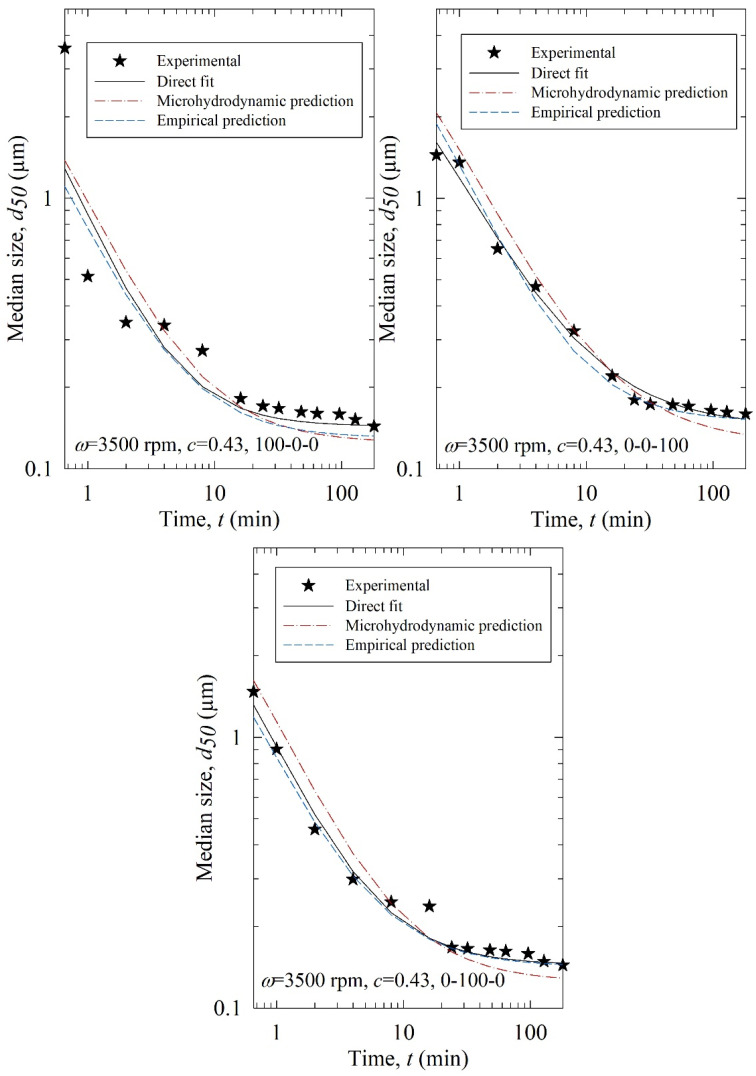
Experimental time-wise evolution of the median particle size for the test runs, their direct fit by the *n*th-order breakage kinetics model, their prediction by the *n*th-order breakage kinetics model augmented with elastic-net regression using the MHD parameters, and their empirical prediction by the *n*th-order breakage kinetics model augmented with a decision tree using the process parameters (empirical prediction).

The fitting parameters of the *n*th-order model, i.e., *k*, *n,* and *d*_lim_, were predicted using (i) the MHD parameters (MHD prediction): *σ*_b_^max^, *α*_b_, *a*, and $\Pi \cdot \sigma_{y}$ and (ii) the process parameters (empirical prediction): *ω*, *c*, and *x*_100_–*x*_200_–*x*_400_. Several machine learning approaches were examined to find the best model (refer to Table S3). For the training data set, leave one out. Cross-validation RMSE values were small and close for some of the approaches, indicating those models are not overfitting and safe to be used [59]. Among those models, the one with the smallest test RMSE was selected: elastic-net regression and decision tree when MHD parameters and process parameters were used as predictors, respectively. The predicted parameters and RMSE of the predicted *d*_50_ are reported in Table 4, and the predicted evolution of the median particle size is illustrated in Figure 11.

According to the RMSEs in Table 4 and the predicted profiles in Figure 11, the process parameter-based predictions were superior to the MHD parameter-based predictions. Except for Run T1 (3500, 0.43, 100-0-0), the process parameter-based (empirical) predictions agreed reasonably well with the experimental data. The poorer performance of the MHD parameter-based predictions originated from the inability of the MHD model to treat poly-dispersed beads, especially bead mixtures, accurately in the training. The MHD model treated bead mixtures as equivalent monodispersed spheres with the average bead size of the constituent beads. Although the beads are almost spherical [20] and narrowly sized (refer to Table 1), they are not monodispersed. As the MHD model was limited to monodisperse beads, its predictions were not successful. Indeed, when the bead size was fixed and no bead mixture was used in an earlier study for the milling of another drug, the MHD parameter-based predictions were superior to the process parameter-based predictions [35]. Here, process parameter-based predictions were reasonably good.

### 3.5. Identification of the Optimal Process–Bead Sizes Based on Merit Scores

**Figure 12:**
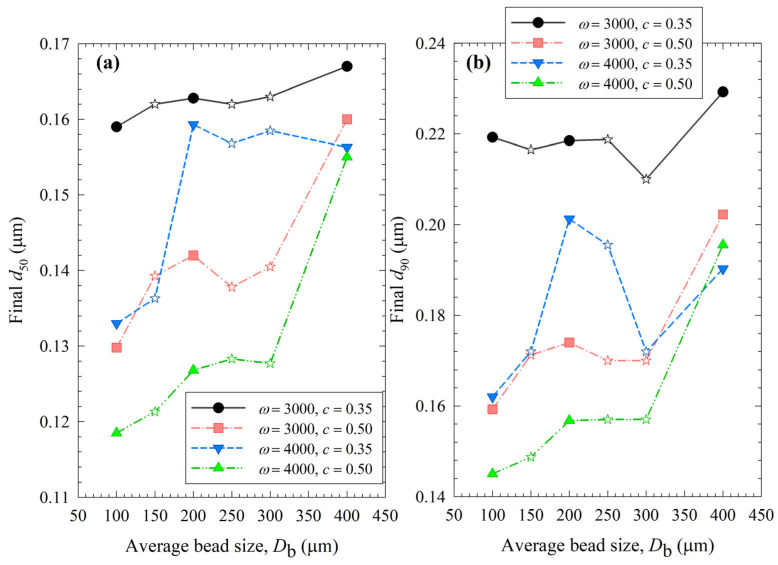
Impact of the average bead size on (**a**) the final median size and (**b**) the final 90% passing size of the milled GF suspensions for each ω–c pair. Average bead sizes of 150, 250, and 300 µm correspond to 50–50% *w*/*w* mixtures of 100–200 µm, 100–400, and 200–400 µm beads, respectively, and those are shown with star symbols.

A quick and rough method for identifying the optimal process is to compare the median size *d*_50_ and 90% passing size *d*_90_ of the final suspensions after 3 h milling, which allows for assessing the capability of the WSMM to produce the finest particles for the specified set of process conditions. Figure 12 illustrates the impact of bead sizes and mixtures on these sizes at different stirrer speeds *ω* and bead loadings *c*. Except for the final *d*_90_ of the least energetic run (*ω =* 3000 rpm, *c* = 0.35), 100 µm beads resulted in the smallest *d*_50_ and *d*_90_ of drug particles at all conditions among all beads (narrowly sized and mixtures). Mixing the beads did not provide a significant synergistic positive impact; all bead mixtures produced coarser drug particles than 100 µm. The results suggest that except for the coarsest beads (400 µm), the WSMM was optimal with a bead loading of 0.50 at 4000 rpm for the production of the finest drug particles. However, producing the finest particles at 4000 rpm may be associated with other issues (see the discussion below); hence, a metric score that considers multiple criteria to assess the feasibility of an WSMM process is warranted.

**Figure 13:**
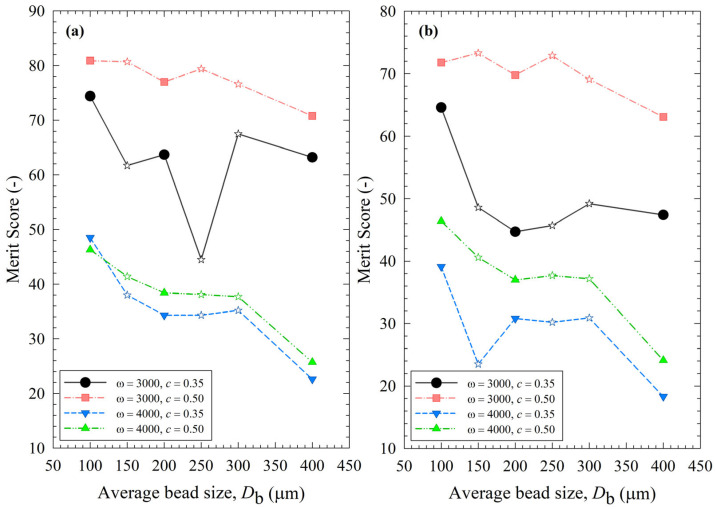
Impact of the average bead size on (**a**) the merit score based on the breakage rate constant, the number of intermittent milling cycles for the median size to reach 0.2 µm, and the power; (**b**) the merit score based on the specific time and the number of intermittent milling cycles for the median size to reach 0.2 µm, and the power for each *ω*–*c* pair. Average bead sizes of 150, 250, and 300 µm correspond to 50–50% *w*/*w* mixtures of 100–200 µm, 100–400, and 200–400 µm beads, respectively, and those are shown with star symbols.

To assess the impact of the process parameters and bead sizes, two merit scores were calculated and presented in Table S2 using Equations (13) and (14). The merit score factors in cycle time through either 1/*k* or *t*_d50_ to reach a median size of 0.2 µm, power *P*, and the extent of heat generation and temperature rise through the number of intermittent milling cycles during *t*_d50_, i.e., *N*_d50_. In general, higher 1/*k*, *t*_d50_, *P*, and *N*_mc_ are not desirable, as indicated by their negative impact on the merit score. Figure 13 depicts the variation of the process merit scores for different processing conditions and average bead sizes. Despite some differences, especially in the impacts of bead mixtures, the impacts of *ω*, *c*, and *D*_b_ on both merit scores followed similar trends. Even a cursory look at Figure 13 immediately reveals that an increase in stirrer speed *ω* from 3000 to 4000 rpm led to a remarkable decrease in the merit scores at both bead loadings due to a simultaneous increase in both *P* and *N*_d50_ (refer to Table S2). Note that *N*_d50_ relates to strict temperature control and the prevention of temperatures exceeding the maximum temperature allowed (35 °C). For non-pharmaceutical products that are not temperature-sensitive, consideration of *N*_d50_ is not warranted as higher maximum temperatures are allowed. Figure 13 also indicates an optimal set of process conditions: *ω* = 3000 rpm and *c* = 0.50. At these optimal conditions, the merit scores were less sensitive to the average bead sizes, and a 50–50% mixture of 100–200 µm beads performed similarly well. Overall, narrowly sized 100 µm beads had the highest merit score. Based on merit score ranking, the bead mixtures did not bring in any significant synergistic benefits because their merit scores were not significantly higher (sometimes worse) than those of the narrowly sized beads.

### 3.6. Overall Assessment and Cost–Bead Wear Considerations

Our experimental results have shown that the bead mixtures did not offer any significant advantages or synergistic effects as compared with narrowly sized beads for WSMM of a drug. We found that the findings and the claims by Patel et al. [32] were mostly applicable to hard inorganic materials because, for the WSMM of griseofulvin, (i) the smallest particles were not obtained by the bead mixtures and (ii) the lowest power consumption did not occur for the bead mixtures. The upshot of our results is that there was no notable advantage to using either bead mixtures or coarser beads alone (200 and 400 µm) except for some ease of handling during the manufacturing operations with the coarser beads and the lower price of the coarser beads. This latter advantage of the coarser beads has been highlighted by Patel et al. [32] for justifying the use of bead mixtures containing fine–coarse beads during the WSMM of non-pharmaceutical, inorganic materials. Unfortunately, an accurate capital cost analysis entails having all the information regarding specific manufacturing operations, the negotiated bead price with the vendors at the manufacturing scale, specific products (parenteral vs. non-parenteral), and the operational culture/best practices of pharmaceutical companies, which is outside the scope of this paper. With all these differences and in the absence of any wear data on bead mixtures, we excluded the cost–wear aspects from the merit score. However, some consideration of these aspects is illuminating.

Table S2 shows the bead prices calculated for each run, and they are inversely proportional to the average bead sizes. However, this simplistic cost analysis misses several important points. First, small beads wear at a lower rate than coarse beads [16]; thus, they have a longer operational lifetime before discarding than the coarse beads. In other words, during manufacturing, the coarse beads would necessitate more frequent replenishment than the small beads. Moreover, small beads are preferable from a wear–product contamination perspective, which is important for pharmaceutical products. To illustrate these points, we present a rough wear–capital cost analysis for our Netzsch Microcer media mill operating with 196 g zirconia beads based on wear data from Li et al. [16] with the same beads used in the current study and similar processing conditions to Runs 1–6. Indeed, some capital cost savings of up to 22% can be made by switching from 100 µm to 400 µm (Table 5), which supports the claims by Patel et al. [32]. Higher savings occur if the beads are to be replaced with new ones on a fixed schedule, such as every month, as a conservative approach in view of the uncertainty in bead wear rates because the wear rate is time-dependent and no data is available for bead mixtures. For example, a 9-year capital cost with monthly replacements without considering the above wear rates would be $11,630, $6620, and $3390 for the 100 µm, 200 µm, and 400 µm beads, respectively. So, replacing the 100 µm beads with the 200 µm and 400 µm beads would lead to 42.9% and 70.8% cost savings, respectively. However, there are many caveats to these cost savings: First, the savings will be lower for the binary mixtures of these narrowly sized beads, e.g., half of the savings for the 50–50% mixtures. Also note that the prices listed in Table 5 are for small quantities of beads from the supplier. For manufacturing scale that entails much larger quantities of beads, we expect that the prices will be lower, and the price differential between the differently sized beads will even be lower. Hence, the capital cost advantage of bead mixtures will be further diminished.

Overall, the only feasible advantage of bead mixtures appears to be some capital cost savings. But this capital cost savings is expected to be much smaller than the operational cost savings when 100 µm beads (faster breakage, lower cycle time; refer to Figure 3 and Table 4) are used. Finally, when in a bead mixture, the small beads may be nipped between the coarse beads, which may increase the wear rate of the beads. In view of this, we purposefully limited the size ratio between the coarsest beads and the smallest beads to 4:1. The use of coarser beads, e.g., 800 µm beads, would have caused a higher extent of damage to smaller beads and was purposefully excluded. The wear in bead mixtures warrants future investigation, as excessive wear can preclude their successful use.

Our results suggest that the smallest beads (100 µm) themselves have all of the benefits related to process–product considerations. However, it is worth mentioning that clogging of the mill screen was observed with the 100 µm beads and their binary mixtures during the first 8–16 min of the respective runs. Therefore, when 100 µm beads are to be used in an industrial-scale operation, pre-milling of the drug particles, e.g., by a rotor-stator mill, can be integrated with the WSMM process. Note that most drug powders, prepared by crystallization processes upstream, warrant size reduction prior to WSMM. Depending on the formulation, the coarse aggregates of the drug particles initially present also act like “big particles,” increase viscosity, and cause clogging. Having an integrated pre-milling step increases the robustness of the WSMM process regardless of whether 100 µm vs. 400 µm beads are used. We suspect that the hydraulic packing and ensuing partial clogging in this study were partly related to the high viscosity of the 7.5% HPC-L-based suspensions (60–120 mPa·s). Without losing good physical stability (see [36]), the use of 1% HPC-SSL may significantly lower suspension viscosity while mitigating partial clogging. Note that even a milled GF suspension with 7.5% SSL–0.05% SDS had an apparent shear viscosity of about 10 mPa·s [60], and the suspension with 1% HPC–SSL would have a much lower viscosity. Another approach to minimizing the clogging issue is to add drug particles to the holding tank of the mill gradually over a period of ~16 min and reduce particle concentration in the milling chamber during the initial phase of the milling when clogging occurs. In a more practical approach, the suspension flow rate can be ramped up from 0–126 mL/min slowly over the first 16 min, which will reduce hydraulic packing effects. In this study with the small-scale mill, multiple flow reversals during the initial phase of milling helped to reduce clogging issues, which obviated the need for these other methods. A practical future study will examine the effectiveness of these different methods to eliminate partial clogging. Finally, a slightly different chamber/screen design by the mill vendor can also help alleviate the hydraulic packing issue.

## 4. Conclusions

In this study, we have assessed the feasibility of bead mixtures (a.k.a. mixed-media or poly-sized media approach) for pharmaceutical WSMM by exploring the impacts of bead size and bead mixtures on the breakage kinetics, the power consumption, and the number of intermittent milling cycles. The breakage kinetics were predicted reasonably well by the *n*th-order kinetic model augmented with decision trees using the process conditions. In general, a stirrer speed of 3000 rpm and a volumetric bead concentration of 50%, along with the smallest beads (100 µm alone), had the highest process merit score. Overall, the bead mixtures did not provide any synergistic improvement except for some savings on the capital cost. The MHD analysis attributed the fastest breakage with the 100 µm beads to the high frequency of drug particle compressions between the beads. Serious deviations from the MHD trends were observed when the model was applied to the bead mixtures, especially at higher energetic conditions. Hence, this study also highlighted the need for the development of a new MHD model that considers polydispersed beads. We conclude that while 200- and 400 µm beads may have some operational ease of handling and lower capital cost, a more detailed analysis suggests these advantages are overrated. Considering lower power consumption and ensuing lower heat generation as well as lower media wear associated with the 100 µm beads as compared with the 200 and 400 µm beads, the driver for utilization of small beads gets even stronger for pharmaceutical applications where product contamination and temperature rise must be minimized. We also conclude that pre-milling of the drug suspension, e.g., in a rotor-stator mill, is required when 100 µm beads are used; however, pre-milling enhances the robustness of any WSMM process regardless of the bead size. Hydraulic packing and partial clogging can be completely eliminated by decreasing the viscosity of the drug suspension, ramping up the suspension flow rate to the target value during the initial phase of milling, adding the drug or the polymer gradually, and/or multiple flow reversals during the initial phase of milling. These practical approaches will be implemented and compared in a future study. The wear from bead mixtures must be thoroughly investigated before such bead mixtures can ever be used for pharmaceutical WSMM.

## Figures and Tables

**Table 1:** Characteristic sizes *d*_10_, *d*_50_, and *d*_90_ of the as-received beads and their size spans.

Nominal Size (µm)	*d*_10_ (µm)	*d*_50_ (µm)	*d*_90_ (µm)	Span ^a^
100	87	112	145	0.524
200	140	194	263	0.633
400	293	405	560	0.659

^a^°$\mathrm{span}=\left(d_{90}-d_{10}\right)/d_{50}$.

**Table 2:** Process conditions used for milling including the mass % of 100–200–400 µm beads.

Run No.	Stirrer Speed (rpm)	Bead Loading (-)	*x*_100_ (%) ^a^	*x*_200_ (%) ^a^	*x*_400_ (%) ^a^
1	3000	0.35	100	0	0
2	3000	0.35	50	50	0
3	3000	0.35	0	100	0
4	3000	0.35	50	0	50
5	3000	0.35	0	50	50
6	3000	0.35	0	0	100
7	3000	0.50	100	0	0
8	3000	0.50	50	50	0
9	3000	0.50	0	100	0
10	3000	0.50	50	0	50
11	3000	0.50	0	50	50
12	3000	0.50	0	0	100
13	4000	0.35	100	0	0
14	4000	0.35	50	50	0
15	4000	0.35	0	100	0
16	4000	0.35	50	0	50
17	4000	0.35	0	50	50
18	4000	0.35	0	0	100
19	4000	0.50	100	0	0
20	4000	0.50	50	50	0
21	4000	0.50	0	100	0
22	4000	0.50	50	0	50
23	4000	0.50	0	50	50
24	4000	0.50	0	0	100
T1 ^b^	3500	0.43	100	0	0
T2 ^b^	3500	0.43	0	100	0
T3 ^b^	3500	0.43	0	0	100

^a^ Mass percentage. ^b^ Test runs that were used to evaluate the prediction capability of the models.

**Table 3:** Statistics of the *n*th-order breakage model fits and the estimated parameters.

Run No.	Run Identifier	*k* (µm^1−n^/min)	*n* (-)	*d*_lim_ (µm)	R^2^	Adj R^2^	SSR
1	3000 0.35 100-0-0	0.292	1.62	0.148	0.992	0.991	0.066
2	3000 0.35 50-50-0	0.191	2.25	0.083	0.965	0.958	0.263
3	3000 0.35 0-100-0	0.214	2.07	0.107	0.978	0.974	0.167
4	3000 0.35 50-0-50	0.116	2.26	0.048	0.960	0.953	0.310
5	3000 0.35 0-50-50	0.248	2.01	0.117	0.976	0.972	0.183
6	3000 0.35 0-0-100	0.210	2.37	0.076	0.954	0.946	0.343
7	3000 0.50 100-0-0	1.54	1.89	0.130	0.969	0.964	0.189
8	3000 0.50 50-50-0	1.46	1.91	0.139	0.968	0.962	0.184
9	3000 0.50 0-100-0	1.39	1.92	0.142	0.973	0.968	0.158
10	3000 0.50 50-0-50	1.18	2.01	0.138	0.980	0.976	0.123
11	3000 0.50 0-50-50	1.28	1.99	0.141	0.990	0.988	0.062
12	3000 0.50 0-0-100	0.85	1.85	0.150	0.996	0.995	0.027
13	4000 0.35 100-0-0	1.22	1.18	0.133	0.890	0.870	0.777
14	4000 0.35 50-50-0	1.17	1.27	0.136	0.938	0.927	0.427
15	4000 0.35 0-100-0	0.571	1.88	0.140	0.981	0.977	0.149
16	4000 0.35 50-0-50	0.511	2.01	0.130	0.986	0.983	0.109
17	4000 0.35 0-50-50	0.808	1.76	0.156	0.997	0.997	0.018
18	4000 0.35 0-0-100	0.497	2.10	0.127	0.976	0.971	0.181
19	4000 0.50 100-0-0	5.10	2.46	0.119	0.996	0.995	0.014
20	4000 0.50 50-50-0	4.65	2.71	0.113	0.995	0.994	0.018
21	4000 0.50 0-100-0	3.08	2.64	0.114	0.994	0.992	0.025
22	4000 0.50 50-0-50	2.42	2.28	0.126	0.997	0.996	0.013
23	4000 0.50 0-50-50	1.87	2.23	0.128	0.995	0.994	0.024
24	4000 0.50 0-0-100	1.53	1.92	0.153	0.990	0.989	0.063

**Table 4:** *n*th-order model parameters for the test runs obtained via direct fit, and predicted with MHD parameters via elastic-net regression, and predicted with process parameters (empirical) via decision tree.

Run Identifier	Approach	*k* (µm^1−n^/min)	*n* (-)	*d*_lim_ (µm)	RMSE (µm)
3500 0.43 100-0-0	Direct fit	1.45	1.78	0.144	0.623
	MHD Prediction	1.17	1.91	0.125	0.604
	Empirical Prediction	1.54	1.89	0.130	0.668
3500 0.43 0-100-0	Direct fit	1.28	1.88	0.144	0.049
	MHD Prediction	0.953	1.92	0.125	0.092
	Empirical Prediction	1.39	1.92	0.142	0.081
3500 0.43 0-0-100	Direct fit	0.814	2.07	0.144	0.066
	MHD Prediction	0.612	2.01	0.125	0.183
	Empirical Prediction	0.854	1.85	0.150	0.120

**Table 5:** Estimated wear rate and capital cost of the 100, 200, and 400 µm zirconia beads over 9-year use with replacements in the Netzsch Microcer media mill operating with 196 g beads.

Bead Size (μm)	Price ($/kg)	Zr contamination in 6 h (μg Zr/g drug) ^a^	Bead Wear Rate (mg/day)	Bead Wear Rate (%mass/day)	Estimated Usable Years of Beads (-) ^b,c^	Capital Cost over 9 Years ($) ^b,c^	Cost Savings by Full Replacement of 100 µm Beads (%) ^b^
100	549.3	307	35.0	0.0178	3.08	323	–
200	312.7	453	51.6	0.0263	2.09	307	5.12
400	160.2	832	94.8	0.0484	1.14	251	22.2

^a^ Based on wear data in ref. [16], adapted with permission from Ref. [16], 2015, Elsevier. ^b^ Assuming replacement when median bead size is reduced by 5% by wear. ^c^ Excludes handling losses.

## Data Availability

The data are contained within the article and its Supplementary Materials.

## Supplementary Materials

The following supporting information can be downloaded at: https://www.mdpi.com/article/10.3390/pharmaceutics15092213/s1, Table S1: Power per unit volume, apparent viscosity, and MHD parameters; Table S2: Inverse breakage rate constant, specific time and number of intermittent milling cycles for median particle size to reach 0.20 µm, power, merit scores, and capital cost of the beads used in each run; Table S3: Root mean squared errors of the machine learning models based on MHD and process parameters; Figure S1: Timewise evolution of the 10% passing drug particle size *d*_10_ during 180 min of milling with various mass fractions of 100-200-400 µm beads at different stirrer speeds *ω*–bead loadings *c*; Figure S2: Timewise evolution of the 90% passing drug particle size *d*_90_ during 180 min of milling with various mass fractions of 100-200-400 µm beads at different stirrer speeds *ω*–bead loadings *c*; Section S1: All supporting data and analysis (except the analysis performed by JMP software version 17.0.0); Section S2: JMP regression report for the dependence of breakage rate constant *k* on stirrer speed (*ω*), bead loading (*c*), and average bead size (*D*_b_); Section S3: JMP regression report for the dependence of average stirrer power per unit volume (*P*_w_) on stirrer speed (*w*), bead loading (*c*), average bead size (*D*_b_), and viscosity; Section S4: JMP regression report for the dependence of viscosity of the nanosuspensions on stirrer speed (*w*), bead loading (*c*), and average bead size (*D*_b_); Section S5: JMP regression report for the dependence of viscosity of the nanosuspensions on their final median particle size (*d*_f50_).

## Abbreviations

Symbols used	
*a*	average frequency of drug particle compressions, Hz
*c*	bead loading or fractional volumetric concentration of the beads
*d*	particle size, m
*D*_b_	average bead size, m
*e*	restitution coefficient
*F*_b_^n^	average max. normal force during collision of two identical elastic beads, N
*g*_0_	radial distribution function at contact
*k*	breakage rate constant in Equation (8), µm^1–n^∙min^–1^
*K*	coefficient obtained from an empirical correlation
*n*	exponent in the kinetic model
*N*_d50_	intermittent milling cycles during *t*_d50_
*N*_mc_	(total) intermittent milling cycles
*p*	probability for a single drug particle to be caught between the beads
*P*_w_	average stirrer power per unit volume, W/m^3^
PSD	particle size distribution
*R*	radius, m
*R*_diss_	dissipation coefficient of the bead
*R*_diss0_	dissipation coefficient when relative motion of the bead–liquid is absent
*t*	milling time, s
*t*_d50_	milling time required to attain a *d*_50_ of 0.2 µm
*u*_b_	average bead oscillation velocity, m/s
*V*_m_	volume of the milling chamber, m^3^
*Y*	Young’s modulus, Pa
*Y**	reduced elastic modulus for the bead–drug contact, Pa
Greek letters	
*α*_b_	radius of the contact circle formed at the contact of two beads, m
*ε*_coll_	energy dissipation rate due to partially inelastic bead–bead collisions, W/m^3^
*ε*_ht_	power spent on shear of milled suspension of the slurry at the same shear rate but calculated (measured) when no beads were present in the flow, W/m^3^
*ε*_m_	non-dimensional bead–bead gap thickness at which the lubrication force stops increasing and becomes a constant
*ε*_tot_	total energy dissipation rate, W/m^3^
*ε*_visc_	energy dissipation rate due to both the liquid–beads viscous friction and lubrication, W/m^3^
*η*	Poisson’s ratio
*θ*	granular temperature, m^2^/s^2^
*µ*_L_	apparent shear viscosity, Pa·s
*ν*	frequency of single-bead oscillations, Hz
Π	energy dissipation rate attributed to the deformation of drug particles per unit volume, W/m^3^
$\Pi \;\sigma_{y}$	pseudo energy dissipation rate, J^2^/m^6^s
*ρ*	density, kg/m^3^
*σ*_b_^max^	maximum bead contact pressure at the center of the contact circle, Pa
*σ*_y_	contact pressure in drug particle when a fully plastic condition is obtained, Pa
*ω*	stirrer (rotational) speed, rpm
*ψ*	volumetric fraction of drug particles in the drug suspension
Indices	
b	bead
f	final
L	equivalent liquid (milled drug suspension)
lim	limiting
max	maximum
min	minimum
p	drug particle
10, 90	10% and 90% passing sizes of the cumulative PSD
50	median (50% passing size) particle size

## Appendix A

Here, the details of MHD model are reported. Wylie et al. [61] gives *R*_diss_ as

(A1) $$R_{\mathrm{diss}}=R_{\mathrm{diss}0}\left(c\right)+K\left(c\right)D_{b}\rho_{L}\theta^{0.5}/\mu_{L}$$

where *ρ*_L_ is the density of the suspension and *K* is a coefficient given by an empirical correlation of bead concentration *c*

(A2) $$K\left(c\right)=\left(0.096+0.142c^{0.121}\right)/{\left(1-c\right)}^{4.45}$$

*R*_diss0_ in Equation (A1) is the dissipation coefficient considering the squeezing of the milled suspension film between two approaching beads and is expressed as follows:

(A3) $$R_{\mathrm{diss}0}\left(c\right)=k_{1}\left(c\right)-k_{2}\left(c\right)\ln\varepsilon_{m}$$

In Equation (A3), *ε*_m_ is the non-dimensional bead–bead gap thickness at which the lubrication force stops increasing and becomes a constant. According to Sangani et al. [62], *ε*_m_ can be taken as 0.003. *k*_1_ and *k*_2_ were computed using

(A4) $$k_{1}\left(c\right)=1+3\sqrt{c/2}+\left(135/64\right)c\ln c+11.26c\left(1-5.1c+16.57c^{2}-21.77c^{3}\right)$$

(A5) $$k_{2}\left(c\right)=cg_{0}$$

The average bead oscillation velocity *u*_b_ and the frequency of single-bead oscillations *ν* were determined using the calculated *θ* and the following expressions:

(A6) $$\;\nu =\frac{24c}{D_{b}}g_{0}\sqrt{\frac{\theta }{\pi }}\;\;\;\;\;\mathrm{and}\;\;\;\;u_{b}=\sqrt{\frac{8\theta }{\pi }}$$

In a comprehensive MHD model, Eskin et al. [39] considered the elastic contact deformation of the beads along with the elastic–perfectly plastic deformation of the particles caught between them. While the beads frequently collide due to their fluctuating motions in a slurry, which are characterized by *θ*, *u*_b_, and *ν*, the beads capture and compress (deform) fine drug particles to be milled. The average maximum normal force *F*_b_^n^ during the collision of two identical elastic beads was calculated using

(A7) $$F_{b}^{n}=1.96{\left(\frac{Y_{b}}{1-\eta_{b}^{2}}\right)}^{2/5}\rho_{b}^{3/5}R_{b}^{2}\theta^{3/5}$$

The probability *p* of a single (drug) particle with radius *R*_p_ being caught between beads was estimated as the ratio of the volume containing the caught particles to the volume of milled drug suspension falling on a pair of the milling beads as follows:

(A8) $$p=0.97\frac{c}{1-c}{\left[\frac{\rho_{b}\left(1-\eta_{b}^{2}\right)}{Y_{b}}\right]}^{2/5}\theta^{2/5}\frac{R_{p}}{R_{b}}$$

Finally, the reduced elastic modulus of the bead–particle contact *Y** was calculated as:

(A9) $$\frac{1}{Y^{*}}=\frac{1-\eta_{b}^{2}}{Y_{b}}+\frac{1-\eta_{p}^{2}}{Y_{p}}$$
