# Supplementary material for: Do Mixtures of Beads with Different Sizes Improve Wet Stirred Media Milling of Drug Suspensions?
Source: Pharmaceutics. 2023 Aug 26;15(9):2213. doi: 10.3390/pharmaceutics15092213 (PMC10535179; doi:10.3390/pharmaceutics15092213)
Supplement: Supplementary file 1 [file pharmaceutics-15-02213-s001.zip › pharmaceutics-2556931-supplementary.pdf]

pharmaceutics

# Supplementary Materials

# Do Mixtures of Beads with Different Sizes Improve Wet Stirred Media Milling of Drug Suspensions?

**Gulenay Guner [1,2], Mirsad Mehaj [1], Natasha Seetharaman [1], Sherif Elashri [1], Helen F. Yao [2], Donald J. Clancy [2] and Ecevit Bilgili [1,*]**

1 Otto H. York Department of Chemical and Materials Engineering, New Jersey Institute of Technology, Newark, NJ 07102, USA
2 Drug Product Development, GlaxoSmithKline, Collegeville, PA 19426, USA
* Correspondence: bilgece@njit.edu; Tel.: +1-973-596-2998

## S1. All supporting data and analysis (except the analysis performed by JMP software).

**Table S1.** Power per unit volume, apparent viscosity, and MHD parameters.

| Run No. | $P_w$ (W/m³) | $\mu_L$ (mPa.s) | $g_0$ (-) | $\theta$ (m²/s²) | $u_b$ (m/s) | $\nu$ (kHz) | $\sigma_b^{max}$ (GPa) | $\alpha_b$ (μm) | $a$ (mHz) | $\Pi \cdot \sigma_y$ (J²/m⁶s) |
|---|---|---|---|---|---|---|---|---|---|---|
| 1 | $4.90 \times 10^5$ | 119 | 5.62 | $1.93 \times 10^{-4}$ | $2.22 \times 10^{-2}$ | 3.30 | 0.846 | 0.357 | 5.53 | $3.15 \times 10^{15}$ |
| 2 | $5.31 \times 10^5$ | 102 | 5.62 | $4.54 \times 10^{-4}$ | $3.40 \times 10^{-2}$ | 3.71 | 1.00 | 0.579 | 6.40 | $5.14 \times 10^{15}$ |
| 3 | $5.73 \times 10^5$ | 104 | 5.62 | $7.70 \times 10^{-4}$ | $4.43 \times 10^{-2}$ | 3.81 | 1.12 | 0.816 | 6.40 | $6.36 \times 10^{15}$ |
| 4 | $5.73 \times 10^5$ | 100 | 5.62 | $1.41 \times 10^{-3}$ | $5.99 \times 10^{-2}$ | 3.87 | 1.26 | 1.22 | 6.23 | $7.86 \times 10^{15}$ |
| 5 | $5.73 \times 10^5$ | 113 | 5.62 | $1.67 \times 10^{-3}$ | $6.52 \times 10^{-2}$ | 3.64 | 1.30 | 1.47 | 5.41 | $7.31 \times 10^{15}$ |
| 6 | $5.73 \times 10^5$ | 93 | 5.62 | $3.61 \times 10^{-3}$ | $9.58 \times 10^{-2}$ | 3.97 | 1.52 | 2.31 | 5.95 | $1.09 \times 10^{16}$ |
| 7 | $8.23 \times 10^5$ | 51.0 | 13.5 | $1.85 \times 10^{-4}$ | $2.17 \times 10^{-2}$ | 11.1 | 0.839 | 0.354 | 34.0 | $1.47 \times 10^{16}$ |
| 8 | $8.23 \times 10^5$ | 66.6 | 13.5 | $2.63 \times 10^{-4}$ | $2.59 \times 10^{-2}$ | 9.68 | 0.900 | 0.519 | 24.9 | $1.24 \times 10^{16}$ |
| 9 | $9.06 \times 10^5$ | 72.6 | 13.5 | $4.26 \times 10^{-4}$ | $3.29 \times 10^{-2}$ | 9.71 | 1.00 | 0.725 | 23.9 | $1.44 \times 10^{16}$ |
| 10 | $8.23 \times 10^5$ | 70.9 | 13.5 | $6.99 \times 10^{-4}$ | $4.22 \times 10^{-2}$ | 9.36 | 1.09 | 1.06 | 21.1 | $1.55 \times 10^{16}$ |
| 11 | $9.06 \times 10^5$ | 72.4 | 13.5 | $1.01 \times 10^{-3}$ | $5.08 \times 10^{-2}$ | 9.71 | 1.18 | 1.33 | 22.0 | $1.87 \times 10^{16}$ |
| 12 | $9.90 \times 10^5$ | 63.8 | 13.5 | $2.20 \times 10^{-3}$ | $7.48 \times 10^{-2}$ | 10.6 | 1.38 | 2.09 | 24.3 | $2.82 \times 10^{16}$ |
| 13 | $1.36 \times 10^6$ | 76.8 | 5.62 | $8.27 \times 10^{-4}$ | $4.59 \times 10^{-2}$ | 6.84 | 1.11 | 0.478 | 20.5 | $2.09 \times 10^{16}$ |
| 14 | $1.41 \times 10^6$ | 81.2 | 5.62 | $1.51 \times 10^{-3}$ | $6.19 \times 10^{-2}$ | 6.75 | 1.28 | 0.736 | 18.8 | $2.44 \times 10^{16}$ |
| 15 | $1.45 \times 10^6$ | 70.4 | 5.62 | $2.85 \times 10^{-3}$ | $8.52 \times 10^{-2}$ | 7.31 | 1.45 | 1.06 | 20.8 | $3.48 \times 10^{16}$ |
| 16 | $1.41 \times 10^6$ | 73.7 | 5.62 | $4.57 \times 10^{-3}$ | 0.108 | 6.98 | 1.59 | 1.55 | 18.0 | $3.64 \times 10^{16}$ |
| 17 | $1.49 \times 10^6$ | 82.1 | 5.62 | $5.84 \times 10^{-3}$ | 0.122 | 6.80 | 1.67 | 1.89 | 16.7 | $3.72 \times 10^{16}$ |
| 18 | $1.57 \times 10^6$ | 107 | 5.62 | $8.50 \times 10^{-3}$ | 0.147 | 6.09 | 1.80 | 2.74 | 12.9 | $3.34 \times 10^{16}$ |
| 19 | $1.78 \times 10^6$ | 27.1 | 13.5 | $7.47 \times 10^{-4}$ | $4.36 \times 10^{-2}$ | 22.3 | 1.11 | 0.468 | 119 | $8.97 \times 10^{16}$ |
| 20 | $1.78 \times 10^6$ | 40.5 | 13.5 | $9.40 \times 10^{-4}$ | $4.89 \times 10^{-2}$ | 18.3 | 1.16 | 0.670 | 78.4 | $6.48 \times 10^{16}$ |
| 21 | $1.91 \times 10^6$ | 30.9 | 13.5 | $2.03 \times 10^{-3}$ | $7.19 \times 10^{-2}$ | 21.1 | 1.36 | 0.991 | 97.5 | $1.10 \times 10^{17}$ |
| 22 | $1.91 \times 10^6$ | 52.0 | 13.5 | $2.16 \times 10^{-3}$ | $7.41 \times 10^{-2}$ | 16.4 | 1.37 | 1.33 | 58.3 | $6.72 \times 10^{16}$ |
| 23 | $1.91 \times 10^6$ | 47.7 | 13.5 | $3.08 \times 10^{-3}$ | $8.86 \times 10^{-2}$ | 16.9 | 1.47 | 1.66 | 59.8 | $7.94 \times 10^{16}$ |
| 24 | $1.99 \times 10^6$ | 59.0 | 13.5 | $4.66 \times 10^{-3}$ | 0.109 | 15.5 | 1.60 | 2.43 | 47.7 | $7.48 \times 10^{16}$ |
| T1 | $7.40 \times 10^5$ | 68.8 | 8.37 | $2.49 \times 10^{-4}$ | $2.52 \times 10^{-2}$ | 6.86 | 0.890 | 0.376 | 17.8 | $9.84 \times 10^{15}$ |
| T2 | $1.16 \times 10^6$ | 124 | 8.37 | $6.47 \times 10^{-4}$ | $4.06 \times 10^{-2}$ | 6.38 | 1.08 | 0.788 | 14.0 | $1.14 \times 10^{16}$ |
| T3 | $1.20 \times 10^6$ | 94.9 | 8.37 | $3.65 \times 10^{-3}$ | $9.64 \times 10^{-2}$ | 7.30 | 1.52 | 2.31 | 15.4 | $2.50 \times 10^{16}$ |

**Table S2.** Inverse breakage rate constant, specific time and number of intermittent milling cycles for median particle size to reach 0.20 μm, power, Merit Scores, and capital cost of the beads used in each run.

| Run No. | 1/k (min/ μm$^{1-n}$) | $t_{d50}$ (min) | $N_{d50}$ (-) | $N_{mc}$ (-) | $P$ (kW) | Merit Score (-)[a] | Merit Score (-)[b] | Price ($) |
|---|---|---|---|---|---|---|---|---|
| 1 | 3.43 | 25.2 | 1 | 1 | 0.047 | 74.4 | 64.6 | 92.3 |
| 2 | 5.24 | 37.7 | 1 | 1 | 0.050 | 61.7 | 48.5 | 72.4 |
| 3 | 4.67 | 40.8 | 1 | 1 | 0.053 | 63.7 | 44.5 | 52.5 |
| 4 | 8.60 | 39.7 | 1 | 1 | 0.053 | 44.5 | 45.5 | 59.6 |
| 5 | 4.04 | 36.2 | 1 | 1 | 0.053 | 67.5 | 49.0 | 39.7 |
| 6 | 4.76 | 38.0 | 1 | 1 | 0.053 | 63.2 | 47.2 | 26.9 |
| 7 | 0.65 | 12.4 | 1 | 6 | 0.073 | 80.9 | 71.5 | 132 |
| 8 | 0.68 | 11.4 | 1 | 8 | 0.073 | 80.7 | 73.0 | 103 |
| 9 | 0.72 | 11.6 | 1 | 7 | 0.080 | 77.0 | 69.7 | 75.0 |
| 10 | 0.85 | 11.7 | 1 | 10 | 0.073 | 79.4 | 72.6 | 85.1 |
| 11 | 0.78 | 12.1 | 1 | 12 | 0.080 | 76.6 | 68.9 | 56.7 |
| 12 | 1.17 | 14.3 | 1 | 11 | 0.087 | 70.8 | 63.1 | 38.5 |
| 13 | 0.82 | 17.4 | 3 | 28 | 0.117 | 48.5 | 39.1 | 92.3 |
| 14 | 0.86 | 19.7 | 5 | 38 | 0.120 | 38.0 | 29.3 | 72.4 |
| 15 | 1.75 | 16.5 | 5 | 46 | 0.123 | 34.3 | 30.7 | 52.5 |
| 16 | 1.96 | 18.3 | 5 | 49 | 0.120 | 34.3 | 30.2 | 59.6 |
| 17 | 1.24 | 15.2 | 5 | 48 | 0.127 | 35.2 | 30.9 | 39.7 |
| 18 | 2.01 | 22.5 | 8 | 61 | 0.133 | 22.6 | 18.2 | 26.9 |
| 19 | 0.20 | 4.62 | 2 | 56 | 0.150 | 46.3 | 46.3 | 132 |
| 20 | 0.21 | 5.71 | 3 | 75 | 0.150 | 41.4 | 40.5 | 103 |
| 21 | 0.33 | 7.12 | 3 | 77 | 0.160 | 38.4 | 36.9 | 75.0 |
| 22 | 0.41 | 6.23 | 3 | 75 | 0.160 | 38.1 | 37.6 | 85.1 |
| 23 | 0.53 | 6.83 | 3 | 78 | 0.160 | 37.7 | 37.1 | 56.7 |
| 24 | 0.65 | 9.65 | 6 | 106 | 0.167 | 25.7 | 24.1 | 38.5 |

[a]Merit score calculated via $1/k$, $P$, and $N_{d50}$. [b]Merit score calculated via $t_{d50}$, $P$, and $N_{d50}$.

**Table S3.** Root mean squared errors of the machine learning models based on MHD and process parameters.

| Model | MHD parameters | | Process parameters | |
|---|---|---|---|---|
| | Train[a] | Test | Train[a] | Test |
| Linear regression | 0.346 | 0.241 | 0.553 | 0.493 |
| Lasso regression | 0.806 | 0.358 | 0.806 | 0.358 |
| Ridge regression | 0.332 | 0.213 | 0.523 | 0.491 |
| Elastic net regression | 0.440 | 0.180 | 0.518 | 0.465 |
| Decision tree | 0.400 | 0.526 | 0.474 | 0.116 |
| Gradient boost | 0.394 | 0.361 | 0.508 | 0.179 |
| Random forest | 0.414 | 0.308 | 0.489 | 0.198 |
| k-nearest neighborhood | 0.382 | 0.242 | 0.478 | 0.192 |

[a]RMSE of the leave-one-out cross validated predictions are reported.

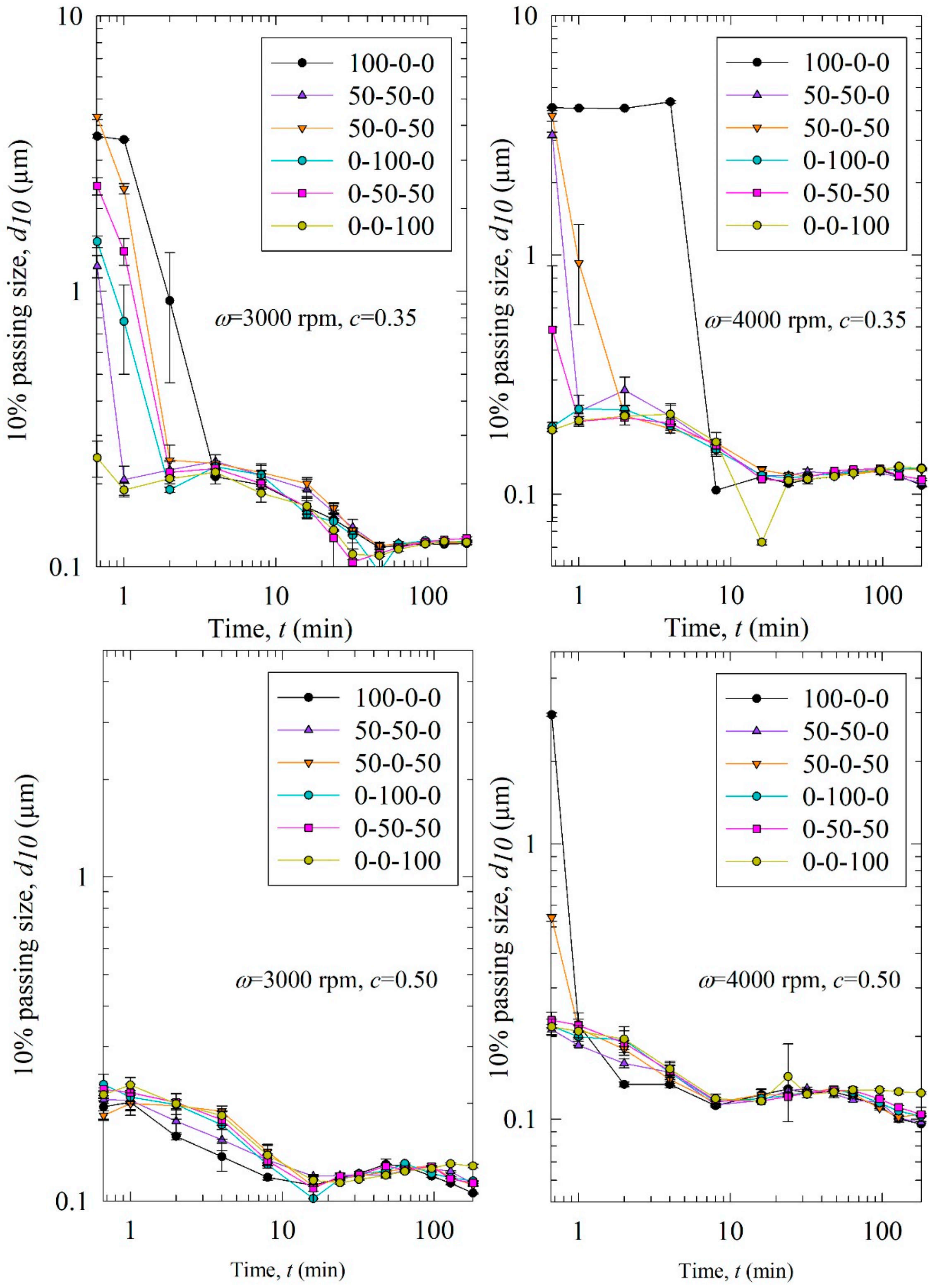


**Figure S1.** Timewise evolution of the 10% passing drug particle size $d_{10}$ during 180 min of milling with various mass fractions of 100-200-400 μm beads at different stirrer speeds $\omega$–bead loadings $c$.

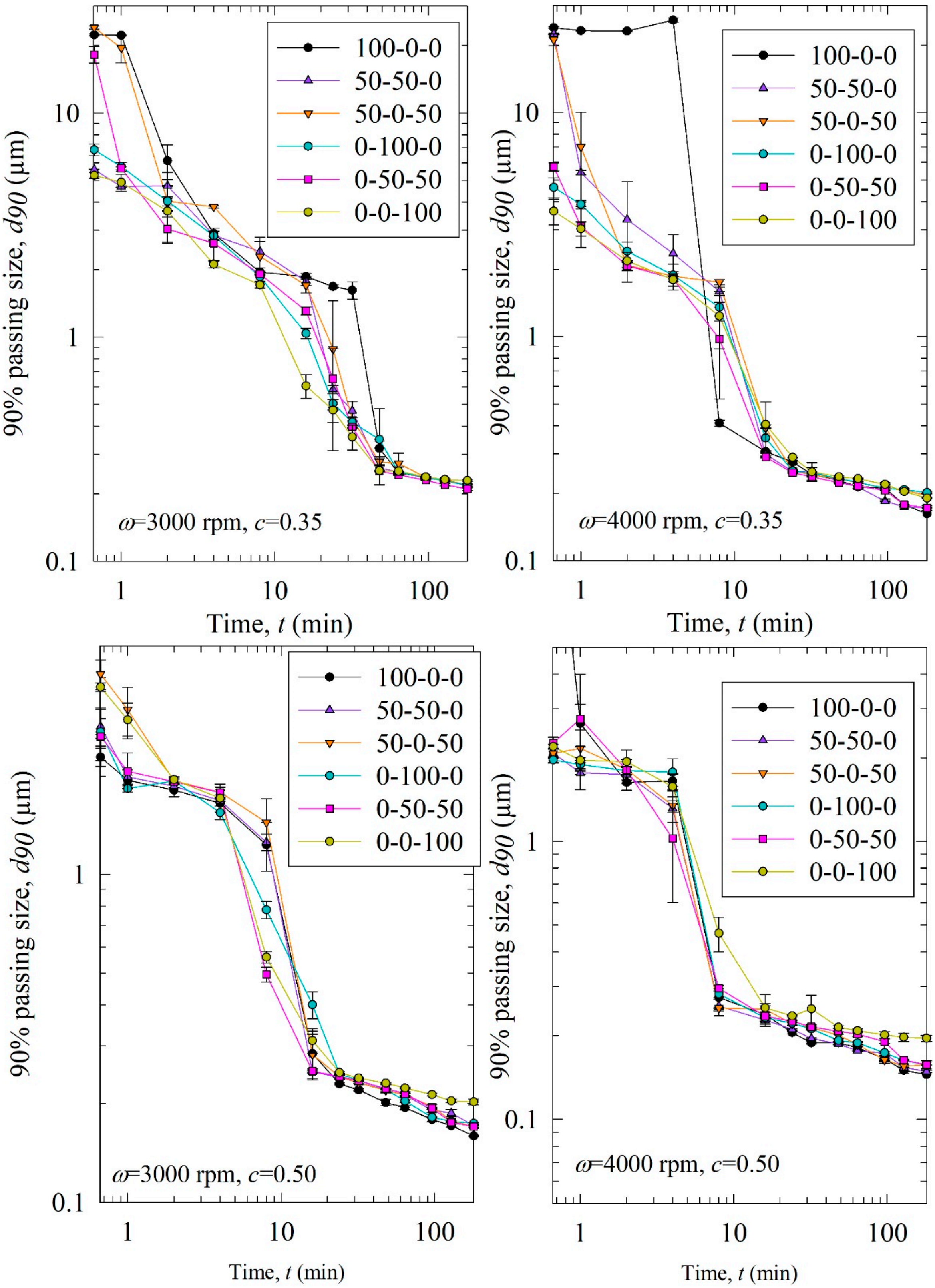


**Figure S2.** Timewise evolution of the 90% passing drug particle size $d_{90}$ during 180 min of milling with various mass fractions of 100-200-400 μm beads at different stirrer speeds *ω*–bead loadings *c*.

## S2. JMP regression report for the dependence of breakage rate constant $k$ on stirrer speed ($w$), bead loading ($c$), and average bead size ($D_b$)

### Whole Model Actual by Predicted Plot

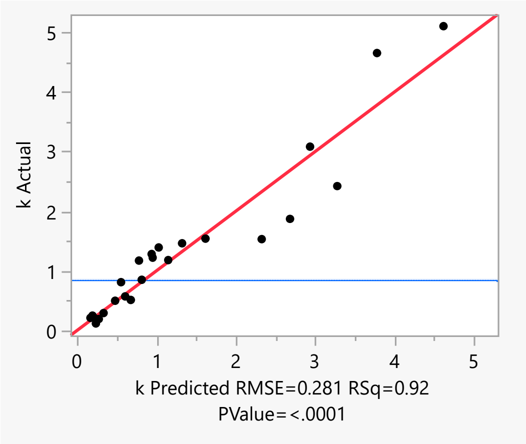


### Effect Summary

| Source | Logworth | PValue |
|---|---|---|
| Log(BeadLoading) | 159.616 | 0.00000 |
| Log(RPM) | 110.146 | 0.00000 |
| Log(Db (mm)) | 37.115 | 0.00000 |

### Summary of Fit

| | |
|---|---|
| RSquare | 0.923995 |
| RSquare Adj | 0.923308 |
| Root Mean Square Error | 0.28097 |
| Mean of Response | -0.15459 |
| Observations (or Sum Wgts) | 336 |

### Analysis of Variance

| Source | DF | Sum of Squares | Mean Square | F Ratio |
|---|---|---|---|---|
| Model | 3 | 318.62888 | 106.210 | 1345.379 |
| Error | 332 | 26.20942 | 0.079 | **Prob > F** |
| C. Total | 335 | 344.83830 | | <.0001* |

### Parameter Estimates

| Term | Estimate | Std Error | t Ratio | Prob>\|t\| |
|---|---|---|---|---|
| Intercept | -28.82373 | 0.870937 | -33.10 | <.0001* |
| Log(RPM) | 3.6457431 | 0.106563 | 34.21 | <.0001* |
| Log(BeadLoading) | 4.4253755 | 0.08595 | 51.49 | <.0001* |
| Log(Db (mm)) | -0.49435 | 0.033738 | -14.65 | <.0001* |

Note: * indicates statistically significance: p-value < 0.01 (orange color) and 0.01 ⩽ p-value < 0.05 (red color); * was not shown for lack of significance, i.e., p-value ⩾ 0.05 (black color in the probability column).

## S3. JMP regression report for the dependence of average stirrer power per unit volume ($P_w$) on stirrer speed ($w$), bead loading ($c$), average bead size ($D_b$), and viscosity

### Response Log(Pw)

### Whole Model, Actual by Predicted Plot

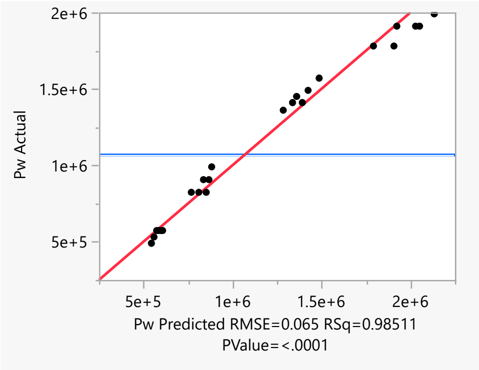


### Effect Summary

| Source | LogWorth | PValue |
|---|---|---|
| Log(w) | 14.223 | 0.00000 |
| Log(c) | 6.147 | 0.00000 |
| Log(Db) | 1.853 | 0.01403 |
| Log(viscosity) | 0.338 | 0.45912 |

### Summary of Fit

| | |
|---|---|
| RSquare | 0.98511 |
| RSquare Adj | 0.981976 |
| Root Mean Square Error | 0.065042 |
| Mean of Response | 13.88355 |
| Observations (or Sum Wgts) | 24 |

### Analysis of Variance

| Source | DF | Sum of Squares | Mean Square | F Ratio |
|---|---|---|---|---|
| Model | 4 | 5.3178261 | 1.32946 | 314.2611 |
| Error | 19 | 0.0803780 | 0.00423 | **Prob > F** |
| C. Total | 23 | 5.3982041 | | <.0001* |

### Parameter Estimates

| Term | Estimate | Std Error | t Ratio | Prob>\|t\| |
|---|---|---|---|---|
| Intercept | -11.01764 | 1.278806 | -8.62 | <.0001* |
| Log(w) | 3.0824606 | 0.140627 | 21.92 | <.0001* |
| Log(c) | 1.1182391 | 0.15444 | 7.24 | <.0001* |
| Log(Db) | 0.0900235 | 0.033277 | 2.71 | 0.0140* |
| Log(viscosity) | 0.06392 | 0.084588 | 0.76 | 0.4591 |

Note: * indicates statistically significance: p-value < 0.01 (orange color) and 0.01 ⩽ p-value < 0.05 (red color); * was not shown for lack of significance, i.e., p-value ⩾ 0.05 (black color in the probability column).

## S4. JMP regression report for the dependence of viscosity of the nanosuspensions on the stirrer speed ($w$), bead loading ($c$), and average bead size ($D_b$)

**Response Log(viscosity)**

**Whole Model, Actual by Predicted Plot**

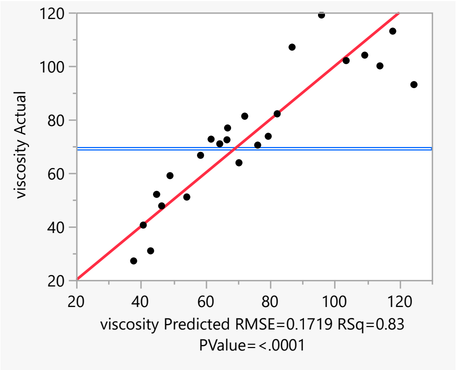


### Effect Summary

| Source | LogWorth | PValue |
|---|---|---|
| Log(c) | 7.041 | 0.00000 |
| Log(w) | 4.303 | 0.00005 |
| Log(Db) | 1.614 | 0.02432 |

### Summary of Fit

| | |
|---|---|
| RSquare | 0.831127 |
| RSquare Adj | 0.805796 |
| Root Mean Square Error | 0.171936 |
| Mean of Response | 4.238615 |
| Observations (or Sum Wgts) | 24 |

### Analysis of Variance

| Source | DF | Sum of Squares | Mean Square | F Ratio |
|---|---|---|---|---|
| Model | 3 | 2.9098542 | 0.969951 | 32.8108 |
| Error | 20 | 0.5912397 | 0.029562 | **Prob > F** |
| C. Total | 23 | 3.5010939 | | <.0001* |

### Parameter Estimates

| Term | Estimate | Std Error | t Ratio | Prob>\|t\| |
|---|---|---|---|---|
| Intercept | 12.05937 | 2.0387 | 5.92 | <.0001* |
| Log(w) | -1.254273 | 0.243994 | -5.14 | <.0001* |
| Log(c) | -1.599659 | 0.196797 | -8.13 | <.0001* |
| Log(Db) | 0.1881845 | 0.077249 | 2.44 | 0.0243* |

Note: * indicates statistically significance: p-value < 0.01 (orange color) and 0.01 ⩽ p-value < 0.05 (red color); * was not shown for lack of significance, i.e., p-value ⩾ 0.05 (black color in the probability column).

## S5. JMP regression report for the dependence of viscosity of the nanosuspensions on their final median particle size ($d_{f50}$)

### Response Log(viscosity)

### Whole Model, Regression Plot

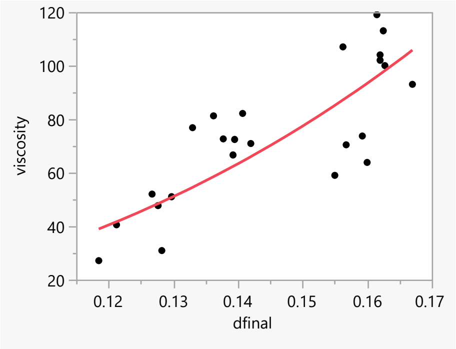


### Summary of Fit

| | |
|---|---|
| RSquare | 0.652295 |
| RSquare Adj | 0.63649 |
| Root Mean Square Error | 0.235232 |
| Mean of Response | 4.238615 |
| Observations (or Sum Wgts) | 24 |

### Analysis of Variance

| Source | DF | Sum of Squares | Mean Square | F Ratio |
|---|---|---|---|---|
| Model | 1 | 2.2837446 | 2.28374 | 41.2720 |
| Error | 22 | 1.2173493 | 0.05533 | **Prob > F** |
| C. Total | 23 | 3.5010939 | | <.0001* |

### Parameter Estimates

| Term | Estimate | Std Error | t Ratio | Prob>\|t\| |
|---|---|---|---|---|
| Intercept | 9.8707802 | 0.878008 | 11.24 | <.0001* |
| Log(dfinal) | 2.9107467 | 0.453082 | 6.42 | <.0001* |

Note: * indicates statistically significance: p-value < 0.01 (orange color) and 0.01 ⩽ p-value < 0.05 (red color); * was not shown for lack of significance, i.e., p-value ⩾ 0.05 (black color in the probability column).
